# Assessing factors that influence graduate student burnout in health professions education and identifying recommendations to support their well-being

**DOI:** 10.1371/journal.pone.0319857

**Published:** 2025-04-15

**Authors:** Jacqueline M. Zeeman, Emili B. Anderson, Isabel C. Matt, Michael B. Jarstfer, Suzanne C. Harris

**Affiliations:** 1 Division of Practice Advancement and Clinical Education, UNC Eshelman School of Pharmacy, University of North Carolina at Chapel Hill, Chapel Hill, North Carolina, United States of America; 2 UNC Eshelman School of Pharmacy, University of North Carolina at Chapel Hill, Chapel Hill, North Carolina, United States of America; 3 Division of Chemical Biology and Medicinal Chemistry, UNC Eshelman School of Pharmacy, University of North Carolina at Chapel Hill, Chapel Hill, North Carolina, United States of America.; Bangkok Thonburi University: Bangkokthonburi University, THAILAND

## Abstract

**Background:**

While well-being has become increasingly important in graduate education, limited information exists regarding factors contributing towards burnout and well-being in Pharmaceutical Sciences Doctor of Philosophy (PhD) students. This exploratory story aimed to identify factors influencing well-being and burnout in these students as well as identify recommendations to support their well-being.

**Methods:**

A two-stage sampling approach was used: (1) purpose sample of Pharmaceutical Sciences PhD students at a public university were invited to participate in a semi-structured focus group or interview to explore factors contributing to PhD student burnout and well-being as well as solicit suggestions for strategies to improve their well-being; (2) Stratified sampling was used to assign participants into focus groups by All-But-Dissertation (ABD) status (i.e., pre-ABD candidates, ABD candidates) to explore experiences that may be unique to these students. Inductive coding and thematic analysis were used.

**Results:**

Six PhD candidates participated in three sessions: three Pre-ABD candidates in one focus group, two ABD candidates in one focus group, and one ABD candidate in one interview. Participants identified relationships and aspects of curriculum and research (i.e., program design, completing milestones) as factors influencing their well-being. Factors influencing participants’ burnout included curriculum and research stressors (e.g., cumulative exams, competing academic and research responsibilities), working overtime, unrealistic expectations, lack of work life balance, and financial burden. While relationships and curriculum and research were factors in both subgroups, unique aspects within these themes emerged between Pre-ABD and ABD participants. Participant recommendations to foster PhD student well-being included improving financial support and encouraging PhD connections.

**Conclusions:**

This study advances knowledge on factors influencing PhD student well-being and burnout, providing suggestions to improve their well-being. Findings highlight curriculum and research factors as well as relationship dynamics influence graduate student burnout and well-being. Findings contribute to broader conversations aimed to support student wellness and reduce burnout in higher education, informing the academy of focused areas and strategies to improve PhD student well-being.

## Introduction

While well-being initiatives have become increasingly important in graduate education to prevent burnout, research on specific factors influencing well-being in Doctor of Philosophy (PhD) students is limited. The Substance Abuse and Mental Health Services Administration (SAMHSA) defines well-being it in eight dimensions: emotional, spiritual, intellectual, physical, environmental, financial, occupational, and social [1]. Well-being encompasses many areas of our lives that are often interconnected; however, low levels of well-being may correlate with increased burnout [2]. Burnout is defined as a syndrome that involves prolonged response to chronic stressors and includes three dimensions [3,4]. The consequences of burnout have been associated with negative personal well-being outcomes as well as negative social and organizational outcomes, including dysfunctional relationships, worsened health, increased substance abuse, higher incidence of depression/suicidal ideation, increased error, and higher turnover rates [4–5].

Originally, burnout was classified as an occupational phenomenon that was most prevalent in careers with intense people interactions, such as healthcare [6]. However, burnout has also been linked to academic training. The risk of burnout within the academic setting has been accompanied by a growing cry from students struggling with mental health concerns. Specifically, PhD students experience higher levels of anxiety, sleep disturbances, depressive symptoms, burnout, and a lower level of well-being compared with students who do not pursue post-graduate education [7,8]. This interconnection is important as burnout can be a cause of a mental health disorder, but mental health conditions can also cause burnout [9]. One study in graduate students found they were six times more likely to experience anxiety and depression compared to the general population [10]. In 2015, the University of Arizona reported that a majority of doctoral students’ experience “more than average stress” or “tremendous stress” [11]. The COVID-19 pandemic has only further exacerbated mental health concerns for this population and research on the well-being of graduate students has greatly increased over recent years potentially due to the pandemic and remote learning [12–14]. This evidence supports a call to action for more research to understand the causes of and to develop strategies and resources to decrease risks of burnout and improve the well-being of graduate students.

Despite literature indicating high levels of stress, mental health, and burnout concerns in PhD students, only more recently has research on factors that affect graduate students’ well-being been reported [15–18]. Surveys in PhD students in medicine and biomedical sciences have identified lack of sleep and basic psychological needs, having at least one current psychiatric disorder, mental health impairment, thoughts of dropping out, challenges in research, pressure to publish, perceived challenges with employment opportunities, limitations in resources, lack of value and poor relationships as stressors or predicters for burnout [8,19,20]. However, resources, supervision, feelings of autonomy and good relationships were seen as factors that influenced resiliency [8,19,20]. One longitudinal survey study indicated that effective mentoring, confidence in the selection of their PhD program, academic development, and sense of belonging were associated with higher mental well-being [21]. Further research confirmed sleep quality and duration, as well as supervisor and peer support, to be positively associated with well-being in graduate students [22,23]. El-Ghoroury et al. [24] found that stressors in psychology graduate students included academic responsibilities, finances/debt, anxiety, and poor work/school-life balance. Whereas coping strategies that improve wellness included support from friends, family, classmates, regular exercise, and hobbies [24]. Barriers to practicing well-being strategies include lack of time and cost [24].

While there has been a growth of studies in graduate student well-being broadly, the research on factors that affect well-being and the influence of unique stressors in graduate training in health professions programs are primarily survey-based. While survey strategies are beneficial for exploring larger audiences, they are limited in their ability to explore detailed subtleties and nuances that provide important context. This is a distinctive population that ultimately represents the future of academia and industry in the United States of America (USA) and beyond; thus, supporting the well-being of this group is warranted and qualitative methods would allow investigators to derive salient themes to identify unique stressors doctoral students face [8]. The research objective of this qualitative study is to address this literature gap by exploring factors that positively and negatively influence well-being and identify recommendations for improving the well-being of Pharmaceutical Science Doctor of Philosophy (PhD) students. Specifically, this study sought to answer the following research questions: (1) what factors positively affect PhD student well-being, (2) what factors negatively affect PhD student well-being and/or cause burnout, (3) what are recommended strategies to improve PhD student well-being, and (4) what other thoughts or suggestions are important to this study? For the purposes of this study, burnout is characterized by prolonged or repeated periods of stress, where a person begins to feel mentally exhausted by their tasks [25]; well-being is characterized as a state of being happy, healthy, and prosperous [26]; and recommended strategies are ideas for action solicited from participants.

## Materials and methods

A two-stage sampling approach was used in this exploratory study: (1) purpose sampling of a public university in the USA (i.e., UNC Eshelman School of Pharmacy) that had previously conducted a quantitative well-being baseline assessment. Results from the baseline assessment indicated that nearly half of the School community was at risk for decreased well-being and burnout – findings similar to many other institutions and disciplines [27–29]. PhD students from this purpose sample were recruited via email from September 19, 2022 to September 30, 2022 to participate in a 60-minute focus group in Fall 2022. Participants provided electronic informed consent through QualtricsXM prior to completing the focus groups. (2) Stratified sampling of PhD students was used to assign participants into focus groups stratified by dissertation status (i.e., All-but-Dissertation (ABD), pre-ABD) to explore experiences that may be similar or unique within these groups. Participants self-identified their status (i.e., ABD, pre-ABD) during focus group signups. Focus groups were led by a doctoral student from a different degree program who was trained in focus group methodology to reduce relational bias (e.g., student-faculty relationship, peer relationship) and to promote a free-exchange of ideas among participants about the PhD student experience.

A semi-structured focus group script was reviewed by faculty and students for refinement to ensure validity and reliability prior to implementation (S1 Appendix). The semi-structured approach allowed for real-time clarification of responses and probing to further understand factors influencing PhD student burnout and well-being, as well as recommendations for strategies to improve PhD student well-being [8,19–24]. Upon completion of focus groups, participants had the opportunity to provide additional thoughts and feedback through an optional, anonymous single-item post-focus group survey. Focus groups were audio recorded and transcribed via Zoom (Version 5.3.11), and transcripts were reviewed for accuracy and de-identified by non-faculty research team members prior to analysis. Transcripts were analyzed using inductive coding. Initial coding and codebook generation was completed by one author. A second researcher coded each transcript independently using the developed codebook. Coded transcripts were compiled and analyzed for agreement. Researchers reviewed all applied codes, discussing any new emerging codes identified and any discrepancies until consensus was reached and data saturation was achieved. Inter-coder agreement was above the accepted 80% threshold for qualitative data [30]. This process ensured validity of code interpretation and richer data analysis. Teams Microsoft Excel (Version 1.6.00.4464) was used to conduct thematic analyses. Theme generation involved grouping codes into overarching patterns or ideas, with subthemes developed for further categorization. Thematic analysis supported relative frequency analysis of themes and subthemes without direct quantification [31]. Ethical consideration by The University of North Carolina at Chapel Hill Institutional Review Board deemed this study exempt on 7 July 2022. (IRB # 21-1629, IRB approval letter is attached). The IRB application specifies no identifiable information (e.g., gender, age, race/ethnicity) will be linked with participant responses. Exploring demographic identifiers, such as gender and age, as factors affecting well-being or burnout was beyond the scope of this study.

## Results

Six PhD candidates participated in three sessions: three Pre-ABD candidates in one focus group (S2 File), two ABD candidates in one focus group (S3 File), and one ABD candidate in one interview (S4 File). There were no responses submitted to the optional, anonymous post-focus group survey. Participants’ duration in the program was well distributed as evidenced by anticipated graduation year: 33.3% expected to graduate in 2023 and 16.7% in each subsequent year through 2027 (i.e., 16.7% in 2024, 16.7% in 2025, etc.) Of the participants, 83.3% identified as White or Caucasian and the majority (83.3%) identified as female.

### Factors influencing well-being

Participants across both Pre-ABD and ABD groups expressed relationships and elements of the curriculum and research as the most prominent factors influencing their well-being (Table 1). Specifically, both groups identified peer relationships as positively influencing their well-being. One participant shared, “not only relationships with other students in [their department] but also the PhD Program more broadly are important for establishing a sense of well-being.”*(ABD participant A2.2, 10/12/22)* When another participant expressed stress associated with the program, they shared “I could only imagine how even more jarring it was for people who didn’t talk to senior students.”*(pre-ABD participant P1.3, 10/13/22)* Participants also discussed having a support dissertation committee as positively influencing their well-being: “My dissertation committee is very supportive, very polite, and always constructive with their feedback, even if they have things they want me to improve. I really like how they’re a source of knowledge and expertise to help guide you. They also help rein it in – like, whoa! This is gonna take way too much time. Or have you thought this? For me, it has been very helpful.”*(ABD participant A1.1, 10/6/22).*

**Table 1:** Well-being themes and sub-themes rank ordered by prevalence.

All PhD Candidate Participants (n = 6)	Pre-ABD Candidates (n = 3)	ABD Candidates (n = 3)
Relationships^#^		
Peer relationships# Support from others who understand the PhD experience^ Supervisor who promotes work-life balance# Dissertation committee support# Both peer and supervisor support# Caring faculty^	Peer relationships Supervisor who promotes work-life balance	Support from others who understand the PhD experience^ Peer relationships Dissertation committee support Caring faculty^
Curriculum and Research#		
Balanced coursework & research in first-year^ Completing tasks/milestones^ Certainty with ABD status^	Balanced coursework & research in first-year^	Completing tasks/milestones^ Certainty with ABD status^
Meaningful Work#		
Fulfillment# Positive feedback^		Fulfillment Positive feedback^
Other		
Work-life balance# Salary increase^ School-level resources^	Salary increase^ Work-life balance	Work-life balance School-level resources^

ABD =  All-but-Dissertation status.

^ Theme or subtheme unique to one subgroup; did not appear in all subgroups.

# Theme or subtheme present across all subgroups. Only themes and subthemes that achieved data saturation within the subgroup are reported.

In addition to relationships, themes regarding aspects of curriculum or research in the PhD program were identified as positively influencing PhD well-being for all participants, although unique elements of the curriculum and research surfaced as prominent themes for each subgroup (Table 1). The first-year program design, specifically the balance and pacing of coursework and research experience, was an aspect of the curriculum and research theme that influenced pre-ABD student well-being, with one participant sharing: “I do think the first-year experience is well-run. It was a good balance between the course work and the research work and everything. I definitely appreciated that.”*(pre-ABD participant P1.1, 10/13/22).* ABD participants discussed how completing tasks or milestones were elements in their curriculum and research that influenced their well-being, along with a degree of certainty with reaching ABD status: “I feel like I have a path, a plan. I’ve got all my specific aims written out. My dissertation committee approved them. I just need to do it. I feel worlds better. I was so stressed going into [preliminary exams] and it’s not that I don’t feel stress now, but I have more clarity.”*(ABD participant A1.1, 10/6/22).*

Additionally, ABD students emphasized the impact of peers and the concept of a shared understanding and support from others who understand the PhD experience as factors positively influencing their well-being (Table 1). One ABD participant shared that “peers are people that understand the most because they’re most likely experiencing the same stressors…so we can empathize with each other and share emotional and mental support for overcoming challenges.”*(ABD participant A2.2, 10/12/22).* Meaningful work was also a theme commonly expressed among ABD participants, with participants emphasizing specifically the impact of positive feedback: “words of affirmation from a PI or lab mate do go a really long way.”*(ABD participant A1.1, 10/6/22).*

### Factors influencing burnout

While elements of curriculum, research, and relationships were noted as factors influencing PhD student well-being, elements across these same domains (i.e., curriculum and research, relationships) were identified as factors influencing PhD student burnout (Table 2). Specific to curriculum and research stressors, participants discussed multiple elements – including balancing curriculum and research responsibilities, cumulative exams, and unrealistic expectations – as specific factors that contributed towards their burnout. One participant shared, “next thing you know you’re starting your first project, taking your [cumulative exams], preparing for your oral [defense]. You really just take off suddenly, and I feel like I wasn’t as prepared as I expected to be.”*(pre-ABD participant P1.3, 10/13/22).* Participants also expressed how the workload often resulted in a need to work more than 40 hours/week. One participant shared: “[students] feel like they have to work significant overtime, or they have to work through their vacation. No one says you have to do this but that’s kind of the unspoken expectation.”*(ABD participant A1.1, 10/6/22).* Participants also discussed how unrealistic expectations within the curriculum and research contributed towards this workload stressor with one participant sharing: “in my first year, no one expected me to really accomplish anything in research because I’m only basically half-time since classes take up the rest of the time. So it’s very bizarre that once you get to [cumulative exams], they’re like ‘oh, now you have to do all of it.”*(pre-ABD participant P1.1, 10/13/22).* Further, inconsistency between mentors or PI expectations was an additional element within curriculum and research stressors that influenced their burnout. One participant elaborated, “there are different expectations [between mentors] and I don’t think that is addressed. For example, there are pretty much no rules [for taking time off]. So, you’re really at the mercy of your PI and how nice they’re feeling or how hard they want to make your life. That’s something I found frustrating.”*(pre-ABD participant P1.1, 10/13/22).*

**Table 2:** Burnout themes and sub-themes rank ordered by prevalence.

All PhD Candidate Participants (n = 6)	Pre-ABD Candidates (n = 3)	ABD Candidates (n = 3)
Curriculum and Research#		
Uncertainty^ Curriculum and research stressors# Balancing curriculum and research# Cumulative exams# Unrealistic expectations# Working overtime# Inconsistency between mentors# Poor training or guidance# Timeline for program completion^	Cumulative exams Curriculum and research stressors Unrealistic expectations Inconsistency between mentors Working overtime	Uncertainty^ Balancing curriculum and research Working overtime Unrealistic expectations Poor training or guidance Timeline for program completion^
Relationships#		
Supervisor relationships^ Supervisor not supporting work-life balance# Poor communication with PI^ Dismissing the student experience^ Lack of understanding about student stress^		Supervisor relationships^ Poor communication with PI^ Dismissing the student experience^ Lack of understanding about student stress^
Work-Life Integration#		
Work-life balance# Impact of pandemic^	Work-life balance	Work-life balance Impact of pandemic^
Financial Burden#		
Low stipend# Stipend inequity#	Stipend inequity#	Low stipend
Other		
Imposter syndrome# Limited and/or expensive parking^ Metrics-based measurement of success#	Limited and/or expensive parking^	Imposter syndrome

ABD =  All-but-Dissertation status.

PI =  principal investigator.

^ Theme or subtheme unique to one subgroup; did not appear in all subgroups.

# Theme or subtheme present across all subgroups. Only themes and subthemes that achieved data saturation within the subgroup are reported.

In addition to curriculum and research factors, participants across both groups discussed how having a lack of work life balance and financial burden contributed towards their burnout (Table 2). One participant stated, “if my [experiment] doesn’t work, I feel it and it keeps me up at night. Even when I leave the school, I’m still thinking about it and it’s hard to stop.”*(pre-ABD participant P1.2, 10/13/22).* Another participant reflected on the financial burden, sharing: “even though we’ve seen a small raise [it is] so far below the amount that would be necessary to provide the same, albeit low, quality life that was provided a couple of years ago in this program [before inflation]. This is something that is causing incredible financial stress to any student who does not have a partner who is making more than them.”*(ABD participant A2.1, 10/12/22).* The stress of a financial burden was discussed further and its significance noted: “it affected people to the point of increasing their willingness to drop out of the program with a master’s or nothing.”*(ABD participant A2.1, 10/12/22).*

In addition to the factors mentioned above, Pre-ABD students noted limited and/or expensive parking as well as stipend inequity between departments as prominent factors affecting their burnout (Table 2). One Pre-ABD student noted that “the student parking pass is the lottery…which is outrageously expensive... then we have to pay an additional fee to get a parking pass for the summer.” *(pre-ABD participant P1.3, 10/13/22).* The Pre-ABD student further explained that the alternative, the park-n-ride, is unrealistic because “you have to plan for two hours for transfers and [travel time]” which is a “big frustration and financial impact” *(pre-ABD participant P1.3, 10/13/22).* Stipend inequity between departments was also specifically noted by Pre-ABD participants, with one explaining: “the stipend is not even the same level as other students in our labs, which is very strange to me. If I had gone through [a different program] and joined the same exact lab, I’d be paid a lot more.”*(pre-ABD participant P1.1, 10/13/22).*

In contrast to the Pre-ABD experience, ABD students emphasized uncertainties in program requirements and expectations around the timeline for program completion as specific elements of curriculum and research that contributed towards their burnout (Table 2). One ABD participant shared, “A big one for me is the lack of transparency and confusion on whether or not you're on the right path or if you were meeting the markers you need to meet…it has a really big negative impact on my well-being.”*(ABD participant A1.1, 10/6/22).* This uncertainty in program requirements, including research progress, translates into feeling burnout given the degree program duration: “You’re constantly worried about [if you’re doing enough because] spending time on one is taking away from time on the other”*(ABD participant A1.1, 10/6/22).* and another sharing “It always feels like you're gonna pay for your time somehow. Because we have requirements to graduate, going slower is maybe better for your day-to-day work-life balance. But then, if you're here for another year or two, that might not be great for your financial well-being, and that can be a stressor that will get you on the other side. So in some ways it can feel like a zero sum game.”*(ABD participant A2.1, 10/12/22).* Additionally, challenges with the supervisor/PI relationship, poor communication with their PI, and others lack of understanding of student stress and/or dismissing the student perspective were relational elements that contributed towards ABD student burnout. One participant described this experience “as a graduate student, you’re immediately shot down, despite multiple students independently having this experience…it makes you feel like you’re not taken seriously and that your experiences aren’t valued”.*(ABD participant A2.1, 10/12/22).*

### Recommended strategies to support PhD student well-being

Participant recommendations for strategies to promote PhD student well-being centered on three primary themes: curriculum and research, financial support, and supportive relationships (Table 3). More specifically, respecting days off was a consistent theme seen across both groups as was ensuring financial compensation was increased to align with minimum living expenses. One participant shared, “look at the cost of living, and make sure the salary offered is aligned – the current stipend does not meet the cost of living for most students.”*(ABD participant A1.1, 10/6/22).* Participants also recommended strategies to improve relationships, including creating more opportunities that encourage PhD connections. For example, one participant recommended “encouraging more cohesion across [departments] – I couldn’t name a single student from [a different department than their own] which is a shame since they’re part of an important research pipeline and we should have an understanding of what they do and I’m sure they would love to understand what we do.”*(pre-ABD participant P1.3, 10/13/22).*

**Table 3:** Recommended strategies to support PhD student well-being themes and sub-themes rank ordered by prevalence.

All PhD Candidate Participants (n = 6)	Pre-ABD Candidates (n = 3)	ABD Candidates (n = 3)
Curriculum and Research#		
Reduce uncertainties / clear expectations & requirements^ Respect days off# Quality over quantity of opportunities^ Provide an unbiased outlet^	Respect days off	Reduce uncertainties / clear expectations & requirements^ Quality over quantity of opportunities^ Provide an unbiased outlet^
Financial Support#		
Increase stipend/compensation# Expand health insurance coverage^ Improve parking access^	Expand health insurance coverage^ Improve parking access^	Increase stipend/compensation
Relationships#		
Encourage connections with other PhD students# Encourage connections with others in the School# Acknowledge student stress^	Encourage connections with other PhD students	Encourage connections with other PhD students Encourage connections with other in the School Acknowledge student stress^
Other		
Create actionable change^ Decrease stigma with seeking mental health support^		Create actionable change^ Decrease stigma with seeking mental health support^

ABD =  All-but-Dissertation status.

^ Theme or subtheme unique to one subgroup; did not appear in all subgroups.

# Theme or subtheme present across all subgroups. Only themes and subthemes that achieved data saturation within the subgroup are reported.

Pre-ABD students, in addition to the suggestions above, specifically recommended improving insurance coverage and parking access to help reduce financial burden (Table 3). For example, one participant suggested “adding dental and vision coverage to the health care plan”*(pre-ABD participant P1.1, 10/13/22),* which was echoed by multiple students, with one emphasizing, “I completely agree with that. As someone who needs glasses…I hope I don’t break them anytime soon, because I definitely can’t do much about it.”*(pre-ABD participant P1.3, 10/13/22).* Pre-ABD participants also recommended improving parking access as a strategy to reduce the financial burden experienced by PhD students: “have parking available for everyone that’s affordable would help.”*(pre-ABD participant P1.2, 10/13/22).*

ABD participant recommendations emphasized an opportunity to create and implement actionable change, reduce uncertainties and communicate expectations regarding the curriculum and research requirements, and encourage connections within the School community (Table 3). ABD students often recommended a concept deemed actionable change, such as a change that goes beyond providing resources or emails and creates change that gets at the root problem. For example, one ABD participant reflected on mental health resources, specifically a recent death by suicide in the undergraduate student population, sharing: “it was just awful and all I ever heard about it was an email. I think we need to take time to recognize that that’s a sign things are not good.”*(ABD participant A1.1, 10/6/22).* Reducing uncertainties within the curriculum and research was also recommended by ABD students, specifically, recommending improved communication about curriculum and research requirements. One ABD participant shared: “sometimes I feel like there’s a list of requirements, and someone somewhere has that list, but I don’t have the list, even though I’m the one supposed to be meeting those requirements.”*(ABD participant A2.2, 10/12/22).* Although peer relationships were identified as a prominent factor improving student well-being, ABD students expressed a desire to also connect with the larger school community. One ABD participant suggested “encouraging connection within the school so that students don’t feel alone because PhD students can feel kind of siloed in what they’re doing and feel disconnected from other students and faculty.”*(ABD participant A1.1, 10/6/22).*

## Discussion

This study adds to the growing literature exploring factors that influence well-being of PhD students. This is an understudied population that has been mostly limited to quantitative data collection, with a few survey studies beginning to explore factors influencing burnout in a broader graduate student population. This research aligns with call to actions for institutional approaches to support well-being and facilitate climates where graduate students can thrive in their intellectual and personal growth [32,33]. Through the utilization of semi-structured interviews and focus groups, participants provided rich insights through descriptions of their experiences related to the well-being of PhD students. This discussion aims to synthesize the factors identified through thematic analysis to provide insight into elements of the PhD student experience that influence their well-being, burnout, and/or both, and thus ultimately inform strategies and recommendations to support PhD student well-being. Insights uncovered through this study fills an existing literature gap by expanding on factors that contribute positively and negatively towards PhD student burnout. Findings can inform actionable recommendations to improve the PhD student well-being and reduce factors influencing their burnout.

### Synthesis of well-being, burnout, and recommended strategy themes

Across both Pre-ABD and ABD groups, themes relating to curriculum and research, relationships, and finances were found to positively impact student well-being (Table 1) as well as contribute towards their burnout (Table 2) and thus were a primary focus area of participants’ recommendations (Table 3). Consistent with the results of this study, previous literature has identified academic responsibilities and challenges in researchers as common stressors for PhD students [19,21,24]. However, this study expands upon this, such that themes associated with curriculum and research not only influence burnout (Table 2) but can also positively influence well-being (Table 1).

Further, while Zhang et al. [21] studied graduate students by class year (i.e., first year, second year, etc.), this study advances prior work by exploring how factors impact PhD students at different stages in their program by dissertation status (i.e., Pre-ABD and ABD). The curriculum and research themes that affected well-being were generally related to the program structure and balance of curriculum and research as they progressed (Table 1). For example, Pre-ABD students noted the structure of the first-year program helped them acclimate to a graduate program, therefore supporting their overall well-being. Furthermore, ABD students shared how progression through the program (e.g., achieving ABD status) provided them greater clarity and confidence, which supported their well-being. However, aspects of the curriculum and research also led to increased levels of burnout (Table 2). Burnout themes relating to balancing curricular and research responsibilities (e.g., cumulative exams and curriculum and research stressors), as well as themes related to individualized experiences within the PhD program, including feelings of uncertainty around program expectations or research progress and working overtime, are consistent with previous studies [19,20,24]. Unique to this study were sentiments of inconsistencies between mentor expectations and how some of aspects of curriculum and research – such as cumulative exams, working overtime, and unrealistic expectations – compounded on each other to influence burnout (Table 2). More research is needed to further explore these emerging themes of positive and negative effects of curriculum, research, and mentorship expectations, and how compounded responsibilities influence burnout in PhD students in health professions programs. Future research should continue to evaluate stressors of PhD students at different stages (e.g., class year, ABD status).

Relationships were also found to influence both well-being (Table 1), burnout (Table 2), and recommendations (Table 3), with unique relationship factors and associated recommendations for well-being emerging in this study that are not reflected in prior studies. Notably, while a positive relationship with a dissertation chair was noted to predict positive well-being in early years of the program [21], this study found a positive relationship between dissertation committees and well-being within ABD students, highlighting the impact of positive relationships from the broader mentorship network (Table 1). Additionally, our study results suggest a connection between relationships and the curriculum and research themes. For example, participants expressed that positive relationships with supervisors, dissertation committees, and peers helped students feel supported through curriculum and research stressors (Table 1). Whereas lack of supporting relationships led to students to feel isolated, dismissed, and uncertain in their research and the curriculum, and thus influenced their burnout (Table 2). In addition to these emerging themes, other findings related to supervisor, peer, and family relationships are consistent with previous literature. Specifically, prior studies [21,23] found that the quality of supervisor support was a significant predictor for PhD student satisfaction, and peer support was also an important predictor for PhD satisfaction [23]. Furthermore, El-Ghoroury et al. [24] found support from friends, family, and classmates were common coping strategies for stress among psychology graduate students. Kusurkar et al. [19] also identified supervision and good relationships as energizers and poor relationships as perceived stressors in PhD work.

Further emphasizing the impact of positive relationships, our study found that PhD students, specifically ABD students who spend most of their time in their research lab, recommended increasing opportunities to form connections with other PhD students outside of their lab or department, as well as members of the broader school community, to improve their well-being (Table 3). It is important that other programs are aware of the impact of positive relationships on PhD student well-being in order to create more opportunities for networking both within and outside their research teams in order to promote a sense of connection and belonging to the full school community.

Although previous literature has identified work-life balance as a predictor for burnout in graduate students, results from this study expands upon this factor to include the connection between supervisor and work-life balance [20,24]. When participants described the impact their supervisor or PI had on their well-being, it was often mentioned in relation to their ability to support work-life balance. Participants shared their well-being was impacted positively when their supervisor promoted work-life balance (Table 1) and negatively when they felt their supervisor did not value it (Table 2). Furthermore, this concept connects to the curriculum and research theme of inconsistencies in mentors’ expectations and working overtime influencing burnout (Table 2). These connections support the Pre-ABD students’ recommendation to respect days off, specifically with University endorsed Well-Being Days (i.e., no classes) (Table 3). The theme of supportive supervisor relationships on improvements in work-life balance (Table 1) is worthy of further exploration across PhD programs at other institutions.

The financial burden PhD students face was a frequent theme that appeared during focus group discussion relating to burnout (Table 2). This theme is consistent with previous literature that identified finances and debt as a common stressor for PhD students [24,33]. All focus group participants expressed that the current stipend for PhD students was inconsistent with the cost of current living, thus influencing their burnout, and this was especially challenging for students without dual income partners. Improving financial support was a notable recommended strategy to better support PhD student well-being (Table 3). Considering financial stress is one of the strongest correlates with graduate student mental health [33], institutions should critically assess stipend amounts in relation to levels of inflation and PhD student needs. Programs should consider regular reevaluation of the stipend amount to assess if it is a livable wage in the current economic climate.

Participants also identified a number of recommended strategies to foster PhD well-being, which aligns with national calls to action to promote graduate student well-being through cultural, organizational, and environmental strategies [8,33]. Participants in this study emphasized the importance of relationships for improving student well-being and identified recommended strategies to enhance these relationships (Table 3). Findings suggest institutions should provide opportunities for PhD students to connect with each other, regardless of department or research group. Further, institutions should promote opportunities to build relationships across all communities (i.e., professional students, graduate students, faculty, and staff), such as informal social events [33] that allow community building outside of the academic setting. Other ideas include more formal networking events and opportunities to present research to larger audiences (e.g., PhD students in other departments, professional students, faculty, and staff).

As curriculum and research themes were found to both increase well-being (Table 1) and influence burnout (Table 2), recommendations to improve the curriculum and research environment were common (Table 3). For example, respecting days-off as a break from the curriculum [33], reducing uncertainties, and communicating expectations [33] were suggested to help improve PhD well-being (Table 3). To help address the recommendations for clarity in expectations, programs could consider focusing energy on creating clear expectations for program requirements and completion. [33] For example, posting expectations on the institution’s website [33] and explicitly reviewing student progress in completing requirements at biannual evaluations with supervisors and reinforcing the graduate student handbook as a guide. However, to reduce uncertainties, ensuring that what is on the website is up to date with current requirements [33].

### Example strategies to support PhD student well-being

Strategies that have been implemented through our institution’s leadership or well-being action plans that align with broader calls to action in PhD students [33] and address many of the participants’ recommendations that other programs could adopt include: providing resources to promote and facilitate mental health [33]; hiring an embedded mental health counselor as an unbiased outlet; identifying and promoting alternative, less cost-prohibitive resources available on the School’s well-being website; and implementing a University-wide increase in the minimum PhD student stipend to better match rates of inflation and living expenses [34]. Across the University, Wellness Days [33] have also been established to create opportunities for protected well-being time in the curriculum (e.g., no class), including for PhD programs. For students that are primarily in labs and which University Wellness Days do not apply, the Pharmaceutical Sciences PhD program now includes a newly added vacation policy [33] outlined in the Student Handbook. While these are positive steps to support PhD well-being, more research and exploration is needed to discover the impact of the stipend increase and strategies to better close the gap between the initiative and implementation of Wellness Days for PhD students (e.g., consistency within PIs to uphold protected time).

To promote connection and build relationships, institutional interventions include: guidance around best practices for the student-advisor relationship [33] outlined in the graduate student Expectations Document; peer-mentors [33] for incoming students and regular wellness and simple social events [33] (e.g., Bagel Mondays) that provide opportunities to leave lab, grab a snack midday and socialize with peers, faculty, and administrators; robust mentor training programs to foster the student-advisor relationship [35]; a pilot “homes’ model across years and sub-disciplines to facilitate a sense of belonging, and support social engagements with a small stipend [33]. These are a select example of strategies that other PhD programs could consider to facilitate mental health access, promote true breaks and work-life balance, cultivate relationships and mentorship, and improve financial support [33,35].

While this exploratory study addresses a critical literature gap, it is limited to one institution and future research should explore the PhD student experience at other institutions. Additionally, focus group participation was voluntary, introducing the possibility of participant bias. To reduce relational bias introduced by a faculty or peer focus group moderator, a student researcher in a different doctoral program moderated the PhD student focus groups to help participants feel comfortable sharing their honest experiences and recommendations without concern or fear of repercussions from individuals who may be viewed as authoritative (e.g., faculty, administration). Lastly, the focus groups were conducted during a two-week period in October, which may not be fully representative of the PhD student experience during other times of the year. Future opportunities exist to conduct longitudinal studies of this population with future research. Additionally, the impacts of COVID-19 on participant well-being was beyond the scope of this study.

## Conclusion

This study advances knowledge on factors that influence PhD student burnout and well-being as well as provides suggested recommendations for strategies to improve their well-being. Participants identified several factors that contribute to PhD student burnout – including curriculum and research stressors, work-life balance, and financial burden – and described positive relationships and aspects of the curriculum and research program structure as factors contributing towards PhD student well-being. Participant recommendations focused on reducing uncertainties and providing clarity on curriculum and research requirements, enhancing financial support including increasing stipend/compensation, and encouraging relational connections with other students and faculty as specific strategies to promote PhD student well-being. This study provides critical insight into factors that influence PhD student well-being and burnout and may inform other programs and guide graduate education more broadly on strategies to support PhD student well-being. Further research should explore how the identified factors influence PhD student burnout and well-being at other programs.

## Supporting information

S1 Appendix Focus group script. (DOCX)

S2 File Focus group transcript 1. (DOCX)

S3 File Focus group transcript 2. (DOCX)

S4 File Focus group transcript 3. (DOCX)

S5 File Codebook definition and Themes-subthemes. (XLSX)
