# Supplementary material for: Assessing factors that influence graduate student burnout in health professions education and identifying recommendations to support their well-being
Source: PLoS One. 2025 Apr 15;20(4):e0319857. doi: 10.1371/journal.pone.0319857 (PMC11999156; doi:10.1371/journal.pone.0319857)
Supplement: S1 Appendix — (DOCX) [file pone.0319857.s001.docx]

| **S1 Appendix. Semi-Structured Focus Group Script** | |
| --- | --- |
| **Time** | **Prompt** |
| ~5 minutes | Thank you for joining me today to discuss PhD student well-being. We greatly appreciate your time. My name is […] and I’m a research team member who will be conducting the focus group. I work alongside [research team member names]. This research project is entitled identifying factors that impact well-being (IRB #21-1629). You received a copy of the informed consent when signing up and I will recap a few points here. The focus group interview will be recorded and all data will be de-identified prior to analysis and dissemination. Discussion and comments shared during the focus group will be identifiable by other focus group participants. Participants are reminded not to disclose any specific comments or dialogue with others outside the focus group. While well-being and burnout domains were evaluated quantitatively in March 2021 on the well-being baseline assessment, which you may have participated in, the purpose of this study is to further explore factors influencing these domains in PhD students. Burnout is characterized by prolonged or repeated periods of stress, where a person begins to feel mentally exhausted by their tasks. Well-being is characterized as a state of being happy, healthy, and prosperous. Do you have any questions before we get started? |
| ~15 minutes | What factors positively affect your well-being? |
| ~15 minutes | What factors negatively affect your well-being and/or cause burnout? |
| ~15 minutes | What recommendations do you have for strategies to improve PhD student well-being? |
| ~5 minutes | What other thoughts or suggestions would you like to share that you think would be important for this study? |
| ~5 minutes | Thank you for participating in this study. Your input is valuable to our community. The findings of this study will help to inform future well-being efforts. If you happen to think of anything else you’d like to share, please use the post-focus group survey link included in the calendar invite. You will be able to anonymously provide additional feedback. |
|  | |
