# Supplementary material for: Assessing factors that influence graduate student burnout in health professions education and identifying recommendations to support their well-being
Source: PLoS One. 2025 Apr 15;20(4):e0319857. doi: 10.1371/journal.pone.0319857 (PMC11999156; doi:10.1371/journal.pone.0319857)
Supplement: S2 File — (DOCX) [file pone.0319857.s002.docx]

**S2 File. Pre-ABD Focus Group Transcript**

10/13/2022

1

00:00:57.770 --> 00:01:03.330

Moderator: Hi, everyone! I think we're waiting on just a few more people to join us, and then we'll get started.

2

00:02:55.750 --> 00:03:06.450

Moderator: Okay, So i'm gonna go ahead and respect you all times, and just go ahead and get started. Um! And hopefully, they'll just join um us in a few minutes.

3

00:03:06.460 --> 00:03:30.579

Moderator: Um, but thank you all for joining me today to discuss Phd. Student well being at pharmacy programs. We greatly appreciate your time and helping us improve our school through this study. My name is [blinded] and I'm, a farm D student who will be conducting the focus group. Today I work along alongside [blinded] and [blinded] As well as [blinded], who oversees this project as our pi

4

00:03:30.590 --> 00:03:39.990

Moderator: Um. This research project is entitled Identifying structures that impact well, being, and our IRB number is at twenty-one, sixteen, twenty-nine

5

00:03:40.000 --> 00:04:01.099

Moderator: uh. We know you received a copy of the informed consent when signing up um, so they will only just briefly recap it. Now. Uh the focus group interview will be recorded, and all data uh will be de-identified prior to analysis and dissemination, discussion and common shared in the focus. Groups will be identifiable by other focus. Group Participants

6

00:04:01.110 --> 00:04:16.379

Moderator: uh participants are reminded not to disclose any specific comments or dialogue with others outside the focus group meeting for the purpose of our discussion Today we will be focusing on domains of well Being and burnout.

7

00:04:16.390 --> 00:04:33.809

Moderator: These domains were evaluated in the march, two thousand and twenty-one well being baseline assessment, and which many of you may have participated in, while the baseline assessment quantified the well-being assessment. The purpose of a study is to further explore the qualitative factors that contribute to these domains.

8

00:04:33.820 --> 00:04:51.179

Moderator: Um, and these domains are defined as um. For example, burnout is characterized by prolonged or repeated periods of stress where a person begins to feel mentally exhausted by their tasks.

9

00:04:51.190 --> 00:04:58.270

Moderator: and well Being

10

00:04:58.340 --> 00:05:04.350

Moderator: and well being is characterized as a state of being happy, healthy, and prosperous.

11

00:05:04.460 --> 00:05:08.639

Moderator: So before we begin, does anyone have any questions?

12

00:05:10.840 --> 00:05:11.840

P1.1: Nope,

13

00:05:13.400 --> 00:05:14.870

Moderator: is that a question?

14

00:05:16.610 --> 00:05:32.560

Moderator: Nope. Okay, Great? Sorry. I think I just heard the Op. And not the the Nope um part Uh. Okay. So the first question is, what factors positively affect your well being and or bring you fulfillment.

15

00:05:41.320 --> 00:05:43.199

P1.2: Do you have some examples.

16

00:05:44.380 --> 00:05:49.020

Moderator: Um: sure. Yeah. So anything that um

17

00:05:49.370 --> 00:05:50.680

Moderator: helps

18

00:05:50.820 --> 00:05:59.210

Moderator: to bring you fulfillment. So is there anything about your work relationships that um bring you fulfillment.

19

00:05:59.450 --> 00:06:01.960

Moderator: Um, And maybe we can just start there.

20

00:06:03.730 --> 00:06:21.299

P1.2: Um: yeah, I I can start, I think, for me, Yeah, definitely during the last um having a good conversation with other lab mates would um help me to uh move my project forward, and that might be one of the to bring up my fulfillment.

21

00:06:21.480 --> 00:06:22.780

P1.2: And

22

00:06:23.080 --> 00:06:25.120

P1.2: uh, what else?

23

00:06:26.390 --> 00:06:39.580

P1.2: Although it's not a lot, and it's the stipen on the increase one thousand this year. I do feel like um some money also bring me feel happy and some fulfillment in a positive way.

24

00:06:39.640 --> 00:06:40.610

Mhm

25

00:06:46.830 --> 00:06:55.620

P1.1: Yeah, I mean, I think it's pretty broad like what that could include. Um. So part of it is definitely the working environment, and like

26

00:06:56.620 --> 00:07:00.950

P1.1: being a good fit with your pi and the rest of the research group,

27

00:07:01.040 --> 00:07:02.879

P1.1: but also just the

28

00:07:03.100 --> 00:07:04.180

P1.1: um.

29

00:07:04.220 --> 00:07:07.539

P1.1: The structure of the program as well has a big impact on that

30

00:07:07.780 --> 00:07:08.810

Moderator: Mhm

31

00:07:09.700 --> 00:07:27.189

Moderator: Hi P1.2 Um. We just began our first question, so i'll repeat it um to you. Um, But i'm going to ask P1.1. Probably a follow up question while you're thinking. Um! But The question is, what factors positively affect your well being and or bring you a fulfillment.

32

00:07:27.200 --> 00:07:46.400

Moderator: Um. So, P1.1, you mentioned, you know, like that the good environment with your pi, and this the structure of the program in general. Can you talk a little bit more about that? What makes um those relationships good? Um! And what that factors of the program um help to

33

00:07:46.410 --> 00:07:49.629

Moderator: encourage your well-being and bring you fulfillment

34

00:07:52.460 --> 00:07:55.750

Moderator: this could also be for anyone. It doesn't have to be just for P1.1.

35

00:07:59.500 --> 00:08:01.600

P1.1: Yeah. I mean, I think there's

36

00:08:02.200 --> 00:08:19.380

P1.1: different groups. All have different expectations in terms of working style and communication and all these things. And I don't think that programs really address these things at all. So it's very much. There's very high variance, and from group to group,

37

00:08:19.590 --> 00:08:20.860

P1.1: whether that

38

00:08:21.280 --> 00:08:24.189

P1.1: you fit well or not well, um!

39

00:08:24.580 --> 00:08:27.669

P1.1: And, for example, like things like taking time off,

40

00:08:27.830 --> 00:08:30.640

P1.1: they're pretty much no rules about this

41

00:08:30.740 --> 00:08:39.320

P1.1: with programs. So you're really sort of at the mercy of your pi, and how nice they're feeling, or how hard they want to make your life. Um!

42

00:08:39.520 --> 00:08:42.499

P1.1: And so that's something that I found frustrating.

43

00:08:43.220 --> 00:08:50.659

P1.1: And usually pi's are accommodating, but at the end of the day it's the it's the student that has to take the initiative then it's like

44

00:08:51.020 --> 00:08:52.800

P1.1: decide to

45

00:08:54.140 --> 00:08:58.270

P1.1: take care of themselves, and I don't think that that's entirely fair.

46

00:09:05.150 --> 00:09:08.230

Moderator: Do you all agree with um, P1.1's thoughts.

47

00:09:11.230 --> 00:09:12.519

P1.3: I'd say, yeah,

48

00:09:12.910 --> 00:09:29.850

P1.2: yeah, I think the School Sounds like thing in our division there for the Phd students. They required us to have uh annual committee meetings, and um, I think it helps a little bit to follow up the process. But um,

49

00:09:29.860 --> 00:09:39.179

P1.2: on the other hand, it's also kind of hard to address um. Other committees, members um questions or comments

50

00:09:39.410 --> 00:09:55.589

P1.2: like to fill up everyone's expectations, and i'll also agree that there's lots of variability between labs. Um, for me, I think uh right now it's fine. I can have weekly meetings with my PI, So I can make sure we are on the same page.

51

00:09:55.600 --> 00:10:06.750

P1.2: Um, But uh, personally, I remember when I joined the program. Um, there is another. I forgot his like. I'm not sure, you know. [blinded]

52

00:10:06.760 --> 00:10:28.479

P1.2: he was here maybe three years ago, and he's like we'll have meetings with us and follow up on how we develop our careers, how we update our linkedin and having our idp the updated it. So now, um, I think that part is gone at this moment.

53

00:10:28.490 --> 00:10:35.379

P1.2: okay. Yeah, no one has follow up on that, too. So I agree that we kind of need to take care of ourselves,

54

00:10:41.920 --> 00:10:45.069

P1.3: I guess, is just as a follow up um.

55

00:10:45.080 --> 00:11:05.039

P1.3: And this is specific to like my experience in the Phd program. But, uh, to kind of reiterate what P1.1 mentioned. I had two very different uh experiences with some of the rotations I did, and the styles of the pi is, which is to be expected. We have a nice, you know, spread of personalities in the division. But um!

56

00:11:05.290 --> 00:11:06.479

P1.3: There was like

57

00:11:06.490 --> 00:11:35.529

P1.3: one of my rotations. The pi super relaxed. He was like very pro work like balance. Um, you know, advocating for the students, you know, asking if, like you're taking on too much. Is this the right speed? You know kind of checking in on the flip side. I did a different rotation, and I was. I was a rotator student in this lab, and the the wellness days or something came up where it's like, you know, your first year. You typically have like days off that are given to you because you're not working full time,

58

00:11:35.540 --> 00:11:42.719

P1.3: and I had asked the other students, and they're like Oh, well, you can take it off. Um, We don't like

59

00:11:42.730 --> 00:11:56.129

P1.3: we don't take it off, because you know that's not given to us, and they're not required to give it to us off. Um, but they're like Well, you're in a rotation. We recommend. You do that because once you join your lab, like, if you join this lab,

60

00:11:56.140 --> 00:12:11.410

P1.3: you would not be given those days, and, like, I don't know it kind of left a sour taste in my mouth where I was like it shouldn't be this way like it. Felt very much like, Take what you can get. But then, after that, like you won't be given a chrome kind of thing, and I just I don't know

61

00:12:11.500 --> 00:12:23.109

P1.3: I don't think that's atypical. And from what P1.1 mentioned as well like, I've definitely known some pis in like my division across the School of Pharmacy that are definitely a little bit more like,

62

00:12:23.170 --> 00:12:43.750

P1.3: you know. Tough love kind of styles where it's like. Sorry, you know, if you want to take a day off, you know, here or there that's fine. But you know, if you want to take a week that's insane like you work to do, You're an employee you have to do X, Y. And Z. So there there is that kind of balance, and i'm not saying It's like that prominent. But I can understand that feeling of like

63

00:12:43.830 --> 00:12:54.230

P1.3: it's on you to take care of yourself, where you don't feel supported by your pi, to to also have the freedom to take the time off and have the work, life balance that you you probably want.

64

00:12:57.460 --> 00:13:17.049

Moderator: Thank you for sharing um. So I think we probably already moved into some. My second question about like the things that negatively affect our well being and lead to burnout. But I kind of want to circle back and maybe talk about um things that help your well being in this like the sphere of work, life, balance like, How do you um

65

00:13:17.210 --> 00:13:20.139

Moderator: help to create that balance in your life?

66

00:13:27.580 --> 00:13:31.790

P1.3: I'm. I'm honestly trying to think if I have work.

67

00:13:32.090 --> 00:13:34.699

P1.3: Um, I will say

68

00:13:34.710 --> 00:13:53.429

P1.3: so. I'm part of the Gso. And it's really nice, because I feel like I can get connected to other students, and I can kind of like gauge things that other people are doing and get invited to things that just kind of like plan events with other people. Um! However, that comes with the cost of

69

00:13:53.540 --> 00:14:06.860

P1.3: you're never dissociated from the lab and lab work if you're always hanging out with lab and left people um so unfortunately I don't think i'm very good at that currently. Um.

70

00:14:06.870 --> 00:14:17.210

P1.3: But luckily we're kind of in a spot where I think the availability of going like on hikes on the weekends and going to the beach is really nice and available to us.

71

00:14:17.220 --> 00:14:36.489

P1.3: Um! And also just getting out of you know the [city] bubble with [city] and [city] seems to be like a really great option for us as well, and I've tried to like Take more advantage of that, as well as you know, kind of doing more healthy lifestyle choices of like going to the gym, or you know, just going for a walk when I feel like I really need to get away from things

72

00:14:43.480 --> 00:14:51.040

Moderator: anybody else have any things that uh they like to do like that, like hobbies or exercise that helps um

73

00:14:51.600 --> 00:14:56.819

Moderator: help you to get away from the um. A lab bubble like P1.2 said.

74

00:14:59.640 --> 00:15:04.949

P1.1: I think she covered most of it. Um, One thing I would add, is um

75

00:15:05.140 --> 00:15:09.729

P1.1: for my experience. I didn't do this very well in undergrad,

76

00:15:09.770 --> 00:15:17.810

P1.1: and it was only after working outside of academia for a few years before going back to school, that I sort of figured out

77

00:15:18.240 --> 00:15:24.309

P1.1: how that was supposed to work, and I don't think that I would have. I think I would still be having problems, if not for that

78

00:15:24.800 --> 00:15:27.440

P1.1: period of like being more of just

79

00:15:28.010 --> 00:15:31.229

P1.1: a typical work environment, and not having

80

00:15:31.590 --> 00:15:34.060

P1.1: this sort of nebulous sense of like

81

00:15:35.090 --> 00:15:38.969

P1.1: working styles being, however, you want, and however extreme that you want,

82

00:15:44.190 --> 00:16:01.170

P1.2: yeah, add one more thing. Um, uh, I try to let my lab and PI know that Sunday will be my time off. So I won't respond to the email, and everyone got used to it. No one was sending their emails on Sunday, and

83

00:16:01.180 --> 00:16:07.729

P1.2: I will know that okay, this is my time off, and other things I can address tomorrow.

84

00:16:10.580 --> 00:16:17.560

Moderator: Nice to setting like clear expectations with your PI has helped you.

85

00:16:18.890 --> 00:16:28.769

Moderator: Can anybody else relate to that? Like having a a break day or an off day, that you've set the expectation with your pi or other uh work Colleagues?

86

00:16:35.390 --> 00:16:40.679

P1.3: i'm gonna say no for me personally, but I think it's a work in progress.

87

00:16:40.850 --> 00:17:09.519

P1.3: Um, and like I mentioned like my pi is really good about that. Um, You won't get emails from him post like six o'clock. He's very big into like that's my family time, you know. Like if it's an emergency, you can contact him. And also on the weekends. I don't have that issue. Luckily, so it's not really on me to have set those boundaries. I think they're already in place, which is really nice, and that's also something that drew me to this lab, and I don't think that's typical in some of the other labs I've had experience in. So

88

00:17:09.530 --> 00:17:10.620

P1.3: um

89

00:17:10.869 --> 00:17:16.129

P1.3: not on my part to have done this, but i'm glad like my PI has set up that expectation.

90

00:17:18.500 --> 00:17:25.460

P1.1: Yeah, I think it's also kind of more difficult in the grad school setting, because, like when I was working

91

00:17:25.780 --> 00:17:26.900

P1.1: uh

92

00:17:27.119 --> 00:17:37.629

P1.1: out in the world, I made the conscious choice to like, not have my work email sent to my phone, and so like I wouldn't see it. And so there's like, If I don't

93

00:17:37.650 --> 00:17:43.859

P1.1: if i'm off, then i'm just not at my work, computer, and I don't have to see it, but with school it's like It's hooked up to all of your

94

00:17:44.430 --> 00:17:51.340

P1.1: other extracurricular stuff, and it's probably on your phone, and it's very hard to like decouple from that as just so

95

00:17:51.610 --> 00:17:54.869

P1.1: such a big chunk of your life.

96

00:17:58.240 --> 00:18:15.979

Moderator: Yeah, I I can relate to that. Um. So I think that'll that'll bridge us into um My next question, and what factors negatively affect your well being and or cause burnout. So, P1.1, I hear you like mentioning just the line being blurred between

97

00:18:15.990 --> 00:18:26.039

Moderator: um like your home life and your school life, and your extracurricular and your work life, etc. Do other people relate to that sentiment?

98

00:18:30.290 --> 00:18:43.440

P1.3: I mean I I can certainly understand that. And uh, I can certainly reiterate that as well in my personal life or I guess, in my experience in the Phd Program. Um,

99

00:18:43.450 --> 00:18:55.370

P1.3: but more so, I think my issue with burnout has been related to um I think my managerial style, and just like the feel of the lab and the other people. Um!

100

00:18:55.910 --> 00:19:15.900

P1.3: So as much as I enjoy not having a micro manager. It comes at the cost of I kind of have to figure everything else out on my own. So it's kind of like, Okay, do this thing, and you're like. I've never done this thing before. Who do I ask? Who do I go to? Where are the supplies? Nobody has information readily available for you. So it's a lot of like

101

00:19:15.910 --> 00:19:39.559

P1.3: you're struggling to even get started because you don't even have the basic supplies to get there. And so you're spending all this time you're getting frustrated. There's no organization so just compounds these issues. And then, when you present, you know, maybe one experiment that you're able to accomplish, because you spent all this time looking at these other things like you're not going to be like. Oh, well, I found this reagent, and then next I did this. It's like those are the things that you're expected to do,

102

00:19:39.570 --> 00:19:53.949

P1.3: but because you had no guidance, no like, you know, I would say support in that aspect I feel like that's contributed a lot to my frustrations. Um, and I don't know that it would necessarily be better for me to be micromanaged. But I definitely feel like

103

00:19:53.960 --> 00:20:05.410

P1.3: in the Phd program. From what I've seen so far, it's a lot of like you're on your own and try and like figure it out on your own. I wouldn't say it's cut throat, but it's kind of edging on that

104

00:20:05.420 --> 00:20:19.490

P1.3: where it's a little bit like. “Oh, Well, I figured it out so like you have to as well to prove yourself” and i'm like that's not the case. Are we not here to help each other? Are we not here to do science together? Why is there so much animosity of like helping each other get along?

105

00:20:19.870 --> 00:20:24.579

P1.3: even if you may have struggled like I don't wish that kind of struggle on someone else.

106

00:20:24.660 --> 00:20:29.269

P1.3: So at least that's that's kind of my feeling and frustrations with the burnout.

107

00:20:34.860 --> 00:20:38.369

P1.2: Yeah, I agree on the part that Um, that's it’s a process.

108

00:20:38.380 --> 00:21:04.569

P1.2: They have their own process. So it's also like you need to figure out your own things. I think that, like first encounters the pros are that everything you figure out is yourself so you could celebrate about it. But if it doesn't work. I feel it and it keeps me up at night. Even when I leave the schools and the buildings, I’m still thinking about it, and it's hard to shut up that thought.

109

00:21:04.580 --> 00:21:06.740

P1.2: And um,

110

00:21:06.750 --> 00:21:26.149

P1.2: I think that for me the burned out is um, usually not only because of the research, but sometimes the research is not going well. And also, there is sometimes family issues, maybe my relationship issues. And when all the things come out at the same time, I did have a burnout moment,

111

00:21:26.160 --> 00:21:44.970

P1.2: and um at the time I try to find some counseling resource, and um I didn't find a good one, and also maybe I know what it is but it also takes time to establish the relationship with the counselors, and also take time to figure out what it is and

112

00:21:44.980 --> 00:21:52.450

P1.2: what they do best. So um, I think that part, maybe. Yeah, I can work more on it.

113

00:22:10.170 --> 00:22:26.980

Moderator: Okay. So I wanted to ask more specifically about how your research and the curriculum um impacts your feelings of well-being or burnout um, etc.

114

00:22:34.650 --> 00:22:39.390

P1.3: I guess I I can only speak on like my division in my program.

115

00:22:39.440 --> 00:22:55.490

P1.3: Um, but I kind of like the setup that they have. So like your first year you're like taking those classes that are basically like refreshers or basics of what you should know. So it kind of gauges like making sure everyone's on the same page um,

116

00:22:55.500 --> 00:23:13.559

P1.3: and after that it it's like all research driven. So I kind of feel like You're going zero to sixty in that sense where it's like your first year. It's like, Oh, okay, You're doing some research kind of like undergrad. You're taking classes, and then that's like full time research. You're in, you know the deep end like you got a sink or swim moment kind of thing,

117

00:23:13.570 --> 00:23:37.119

P1.3: and so I didn't find that the curriculum was negatively affecting my perception on the program, or like my mental health. It was a bit of a balance, I will say, because I similarly to P1.1, like I came from industry. So I came from working. So I was just research full time. So then, going back to like classes and balancing class and research was like a whole other shift for me personally. Um,

118

00:23:37.140 --> 00:23:51.889

P1.3: but I didn't think it was unmanageable, and it was actually kind of nice to have all these like fundamentals reiterated my first year and then, but then after that it's like you. Go back to research full time, and then it's like, But the research is like

119

00:23:51.900 --> 00:24:06.730

P1.3: reading a ton of things you've never read before, and trying to also then do them at the same time. So in that aspect of things I think it's expected to have a lot of struggles your second year, which is kind of what I'm currently experiencing. Um,

120

00:24:06.740 --> 00:24:20.069

P1.3: and like I feel like, yeah, I get down days about my experiments. I'm like i'm a total failure. I don't know what i'm doing like, you know Imposter syndrome times ten but I don't think that's like you know the setup

121

00:24:20.140 --> 00:24:30.900

P1.3: to have me fail. I think it's just like an expected process of going through the research stages to get to, you know, being your full blown Phd self at the end.

122

00:24:42.130 --> 00:24:51.289

Moderator: Do others feel similarly? That

123

00:24:51.640 --> 00:24:58.200

Moderator: it wasn't so much like the curriculum that was a stressor or does anyone disagree?

124

00:24:59.680 --> 00:25:18.400

P1.2: Um: yeah, I think in my division. Um, it's also like first year you're taking classes and doing rotations. And then the second year you start your research, and I think it works well for me. Um, maybe just one thing that I don't like a lot is um in our division. We have

125

00:25:18.410 --> 00:25:32.679

P1.2: It's called Cume, and it's like an accumulation exam. So um! You need to take that every month until you get enough points. It's about maybe eight months, I would say half to a year.

126

00:25:32.690 --> 00:25:48.610

P1.2: And um if I, reading something not related to your research, whatever the professor wanted to read, and then take the exam for two hours. Um, I think that part is kind of stressful, and also because it's not related to your research. So, um,

127

00:25:48.620 --> 00:26:03.519

P1.2: I don't really feel that it's really helpful. I just wonder if in the future it can be more like a monthly journal club. We just discuss it, but it’s not graded so it will be less stressful. But we can still learn something, and also it will

128

00:26:03.530 --> 00:26:10.670

P1.2: maybe not taking lots of time for us out of our research, but not using that information in the future.

129

00:26:17.170 --> 00:26:27.190

P1.3: Yeah, I I completely agree with the cume perspective. It seems like a lot of people don't like them. I think a across the grad schools, but they will get rid of them.

130

00:26:27.570 --> 00:26:28.700

P1.3: Yeah.

131

00:26:29.420 --> 00:26:33.150

P1.3: And i'm actually currently in the middle of it as well, and I was just like

132

00:26:33.450 --> 00:26:44.469

P1.3: it. It like kills a whole week of the month, which, if you add it up. That's like two full months that you're likely to just not be doing research, and it's kind of like

133

00:26:44.600 --> 00:27:05.089

P1.3: an unexpected expectation. Then, to make up for that time by working extra hours, either on the weekend, because after your you know end of your second, you're going to your third year you're having a committee meeting where you have to present your research, your oral defense, you become a candidate like That's a lot of things stacked up so quickly that it's like If you're only doing your forty hours.

134

00:27:05.100 --> 00:27:31.260

P1.3: Well, you're losing, you know, a full weeks worth at least once a month. There's no way you're going to catch up to where you are supposed to be by that time, unless you start working more and more like way above the expectation. And so I completely agree with the cume , you know perspective, and I also don't agree that, like that's expected of us unintentionally like it seems a little sneaky to be like, Oh, okay, great. And then next thing you do is like

135

00:27:31.270 --> 00:27:40.709

P1.3: your oral defense. We have to present all of this in-depth research that you haven't had time to do so a two-edge sword. I think, or double-edge sword, I think, is the phrase.

136

00:27:40.980 --> 00:27:41.820

Yeah,

137

00:27:42.350 --> 00:27:47.740

P1.1: yeah, I can't really speak to that. Obviously, I’m a first year, but

138

00:27:47.910 --> 00:28:03.709

P1.1: in the first year no one expects me to really accomplish anything in research, because i'm only like basically doing half time, because the classes take up the rest of the time. So it's very bizarre that once you get to cumes, they're just like Oh, never mind, you have to do all of it.

139

00:28:09.590 --> 00:28:15.409

Moderator: So it sounds like, maybe some like unrealistic expectations. And um

140

00:28:15.670 --> 00:28:19.700

Moderator: timing issues is um impact your well-being

141

00:28:20.870 --> 00:28:50.820

P1.3: yeah, I think it's for me personally. It's that whole like zero to sixty, like like P1.1 said that your first year you're just like cruising along everyone's like, okay, great. You like, just go for one rotations in the next. Next thing you know, it's like you're in cumes. You're starting your first project. You're now expected to do this. Now you have to prepare for orals. It's just like you really just take off suddenly, and I feel like I wasn't as prepared as I expected to be, even though I talked to other students. But I can only imagine how even more jarring it is for people who didn't talk and weren’t

142

00:28:50.830 --> 00:28:54.800

P1.3: prepared um, speaking to like senior students.

143

00:29:03.030 --> 00:29:17.699

Moderator: I think someone uh changing gears a little bit. Someone mentioned um compensation for work, and that it increased this past year. Can we talk a little bit about that? And how Um,

144

00:29:18.090 --> 00:29:22.199

Moderator: you know the compensation impacts your well-being.

145

00:29:25.520 --> 00:29:31.910

P1.2: Yeah, I saw there's like one thousand increase this year. Um, I would say it's not

146

00:29:31.970 --> 00:29:48.429

P1.2: like a lot of money that I can feel the difference. But it still, let me feel a little bit better, because um, as anyone said, uh lots of time we work over hours. So yeah, so I sometimes do feel like I work a little bit more.

147

00:29:53.220 --> 00:29:54.700

P1.3: Yeah, I

148

00:29:54.800 --> 00:30:00.759

P1.3: I have some strong opinions about this quote on quote increase that they gave us um,

149

00:30:01.020 --> 00:30:09.530

P1.3: and i'm not going to de-rail the conversation based on that. But I, you know I do appreciate that it was advocated for,

150

00:30:09.720 --> 00:30:38.089

P1.3: and that we did receive something. However, to me it feels like crumbs, because that's exactly what it is. It doesn't cover inflation increase. It doesn't cost a rent increase. From the discussions I've had with my fellow grad students. Um! Their rent went up like, you know, a hundred dollars or two hundred dollars. If we do the math, it's about less than eighty dollars pre tax that we received per month, so we're not even covering the increase in just rent.

151

00:30:38.230 --> 00:30:51.490

P1.3: And on top of that food has increased, gases increased. It'll fluctuate, but overall, you know the trends are going up, and as much as you know, they toted th e increase in our stipend is like this huge accomplishment

152

00:30:51.500 --> 00:31:19.889

P1.3: also speaking from previous, or like more senior grad students, they're like we've been talking about this for years. We've been talking about it for years. Our stipend has been the same for years, and so to me. It feels a little bit disingenuous to toted it as this huge accomplishment. When one you've known the struggle for, you know, half a decade and two. You basically gave us nothing in comparison. I mean, something's better than nothing. I agree with that. But also it wasn't the impact that we wanted.

153

00:31:20.430 --> 00:31:21.520

P1.3: So

154

00:31:29.530 --> 00:31:33.799

P1.1: I definitely agree with P1.3. Um. It's kind of

155

00:31:35.660 --> 00:31:37.799

P1.1: especially like compared to

156

00:31:37.840 --> 00:31:41.490

P1.1: like. We're not even it's not even the same level as like

157

00:31:42.070 --> 00:31:43.350

P1.1: students.

158

00:31:43.380 --> 00:31:57.259

P1.1: Other students in our labs sometimes, which is very strange to me. It's like If I had gone through [blinded program] and join the exact same lab, i'd be paid a lot more. It's like that doesn't. You're incentivizing people to go

159

00:31:57.800 --> 00:32:00.060

P1.1: a different route. Um,

160

00:32:00.200 --> 00:32:04.970

P1.1: And then there's also I haven't really experienced this personally. Well,

161

00:32:05.200 --> 00:32:24.989

P1.1: but from what I understand with, when you get external fellowships and other things that, like the pay schedule for that ends up very disjointed and strange, where you can get paid like once a semester or something. Um. So the whole, the whole system of like training us like we're not employees, is very weird, and

162

00:32:25.030 --> 00:32:26.529

P1.1: that leads to a lot of

163

00:32:26.960 --> 00:32:29.149

P1.1: uh burden on us.

164

00:32:35.230 --> 00:32:46.980

P1.2: Um, maybe I can't add a little more about the external fellowship, and also the travel. So um, I do apply some external fellowship and got a little bonus, and I think

165

00:32:46.990 --> 00:33:01.490

P1.2: it does help more. It's like fifteen percent more and um the other part is um the travel. So um I’m in the last year of my graduate hopefully. So. Um, I have some time to go to the conferences.

166

00:33:01.500 --> 00:33:29.389

P1.2: and um. So thanks for my PI, he said on the trip that the accommodation and the air fair is covered. So I do feel that part helps a lot, because travel takes lots of money. But um the the travel um stipend within the school is not that well, I do have some difficulty to get my reimbursement my back, and it takes it. Yeah. Last month it takes lots of time

167

00:33:29.400 --> 00:33:40.049

P1.2: to apply for the reimbursement. I know it's there, but it just takes so many times, and sometimes I feel they kind of suspect me to take some money that I is not.

168

00:33:40.060 --> 00:33:52.420

P1.2: I should have. But um! We have the discussion that I should get the money so um! If the reimbursement system could be more smooth it would also help my wellbeing.

169

00:33:58.570 --> 00:34:17.909

Moderator: I think this is um a great segue into my next question, which would be, What recommendations would you suggest to improve graduate student well-being at the school, so I think I heard like compensation. Um, and a smoother reimbursement process. Um,

170

00:34:18.070 --> 00:34:23.660

Moderator: Any thoughts, maybe starting there and then moving to other suggestions.

171

00:34:32.469 --> 00:34:34.090

P1.1: We don't have

172

00:34:34.190 --> 00:34:36.979

P1.1: dental and vision coverage on health care, right?

173

00:34:37.870 --> 00:34:39.249

P1.1: That would be nice.

174

00:34:43.940 --> 00:34:53.119

P1.3: Yeah, I I completely agree with that. Um. And as someone who needs glasses and everything I was like well, I hope I don't break these anytime soon, because uh

175

00:34:53.489 --> 00:34:56.519

P1.3: definitely can't do much about it. Um,

176

00:34:56.550 --> 00:35:10.750

P1.3: and in addition to the general compensation uh, so I have to travel to campus because I live off campus, and the student parking past is the lottery that we have to like go into. Um.

177

00:35:10.760 --> 00:35:20.240

P1.3: It gives you a lot of options, but they're outrageously expensive, like we're talking like five hundred dollars for the academic school year, and then

178

00:35:20.310 --> 00:35:39.340

P1.3: to only make it worse during the summer, when we're still required to work through the summer, our parking passes stop the last day of classes. So then we have to pay an additional fee to get a parking pass for the summer, which is an additional like. I think I pay like a hundred and seventy dollars for, like

179

00:35:39.350 --> 00:35:42.539

the middle of May, through the end of July.

180

00:35:42.560 --> 00:36:03.779

P1.3: And so if you, if you calculate that up, I mean, that's a ridiculous amount. I mean, that's a rents worth amount. They're just paying for parking passes and for students who don't live on campus. I think that's a little bit ridiculous to have us work through the summer as full time employees. But then, d on't provide us the accessibility to travel to campus like

181

00:36:03.790 --> 00:36:21.019

P1.3: you know, you could do the parking ride. But let's be honest. It's not the best alternative, if you have to plan for like two hours of, you know, like transfer in between like It's just a little bit unrealistic, and I find that to be a big frustration and a big financial impact on me. Um,

182

00:36:21.220 --> 00:36:29.609

P1.3: even though you know we're still required to be here and there. Isn't anything that's being done about it, uh, at least for students who travel.

183

00:36:43.400 --> 00:37:09.319

P1.2: Yeah, I also live off campus and needed to find a parking pass.It’s a lot of mondey and even sometimes you can’t get into a the lottery. It's also a stressful process. But I was thinking, maybe, like school pharmacy can have at least maybe several slots for um students, and sometimes, I think it doesn't need to be every day. Like sometimes we have experiments in the early morning, and maybe I just need to have a parking space for several says in that month. So if we have something like available for everyone, and

184

00:37:09.330 --> 00:37:16.409

P1.2: um it’s also affordable, and maybe it doesn't need to be a lot, but it would also help.

185

00:37:19.320 --> 00:37:23.990

P1.1: Yeah, I definitely agree with all of that. Um. And also like,

186

00:37:24.080 --> 00:37:26.030

P1.1: you know, this is what the

187

00:37:26.690 --> 00:37:42.690

P1.1: the transit system is supposed to be for, and It's pretty good in certain parts of town, but you know, when I was when I was moving here I was like. Well, I don't want to have to deal with the parking thing, so i'm gonna live near a bus stop. Well, that's the expensive place to live. So it doesn't really help.

188

00:37:42.700 --> 00:37:46.879

P1.1: I mean, yeah, I don't have to go through those hoops. But then it's like I, Whatever I would have

189

00:37:47.020 --> 00:37:52.019

P1.1: had to pay for parking. I just pay for a more expensive apartment, so it's not really any better.

190

00:37:58.720 --> 00:38:02.879

Moderator: Any other recommendations that you would suggest to the school?

191

00:38:04.910 --> 00:38:13.889

P1.3: Um, so I don't know how realistic this is, and I feel like It's a top-down kind of uh request, if possible, but

192

00:38:14.090 --> 00:38:23.459

P1.3: something that I've talked with fellow grad students in the school pharmacy is, you know, as we discussed, I think, earlier in this meeting.

193

00:38:23.470 --> 00:38:40.019

P1.3: Not every PI kind of considers days, you know, equally of equal weight, and something that I feel like would be worthwhile advocating for and kind of stressing is, we have these wellness days, which you know, we get emails about. We have posters about,

194

00:38:40.340 --> 00:38:57.449

P1.3: but the reality is, unless PIs are like, required to give us those days off like we're not getting them. You know the ones that are a little bit more relaxed about it. A little bit more hands off and like, uh, maybe more like pro student health

195

00:38:57.640 --> 00:39:12.979

P1.3: like they will, you know, Say, hey, if you need to take the time off, take the time off, but otherwise it's kind of defaulted to you're still expected to work It doesn't matter what day it doesn't matter what the school designates it as. And I just feel like maybe I wouldn't say pressuring,

196

00:39:13.010 --> 00:39:14.520

P1.3: but maybe like

197

00:39:14.870 --> 00:39:19.130

P1.3: advocating for the PIs to be more um

198

00:39:19.740 --> 00:39:47.019

P1.3: wouldn't say relaxed, but essentially just like more proactive in giving those students the days off. They're not a lot of days at all. Like we get, maybe like two days a semester, but I feel like that would make a huge difference if you know your PI walks in and is like, hey? It's a wellness day tomorrow. Take the day off. Go do something. Go, walk in a park. Do something for yourself. I feel like that would make a huge impact for at least the grad students um in my division. To feel like their PI, you know is is proactive in their mental health.

199

00:39:50.820 --> 00:40:03.219

P1.1: I definitely agree with that. Um, yeah, I think sometimes it's not even the pi. It's just like, well no one else is taking the day off and i'm behind> so it's just sort of changing the default from

200

00:40:05.110 --> 00:40:07.819

P1.1: I’m not having the day off, to I’m having the day off.

201

00:40:16.240 --> 00:40:18.259

Moderator: What do you think, Um

202

00:40:18.520 --> 00:40:24.179

Moderator: is successful, that the school is doing currently to improve well, being,

203

00:40:27.910 --> 00:40:32.509

Moderator: or like what needs are still not being met.

204

00:40:33.550 --> 00:40:40.179

P1.1: i'll just say briefly, I do. I do think that the first year experience is is pretty well

205

00:40:40.510 --> 00:40:49.400

P1.1: run, at least in my division. Um, I think that they have a good balance between the course work and the research work and everything. Um.

206

00:40:49.660 --> 00:40:52.000

P1.1: And I definitely appreciated that.

207

00:40:57.750 --> 00:41:27.450

P1.3: Yeah, I I agree with P1.1's sentiment. Um, i'm only in my second year. So my first year wasn't that long ago, and I think in general we're a pretty small group coming in, so I felt like the camaraderie was built. And like just my general interactions with other students like, I think our division does build that pretty well. You're only you know group of five people. And then, you know, there's another group of five people who just did the same thing, you guys did, you know, and and just having those interactions.

208

00:41:27.620 --> 00:41:28.919

P1.3: Um!

209

00:41:29.000 --> 00:41:34.640

P1.3: What I don't think is as well done is mingling among the divisions

210

00:41:34.700 --> 00:42:03.589

P1.3: and kind of integrating us all together like we're all under the school of pharmacy. But even under the four divisions like you may have, I think, one class in the fall semester, and then one class in the spring semester together, and then after that it's like you never existed in the same place together, like. I couldn't name you a single [Division/Department blinded] student. Like I couldn't name you any of those other folks which is a shame, because you know, like they're part of this important research, pipeline, and like we should kind of like,

211

00:42:03.600 --> 00:42:19.440

P1.3: you know, have an understanding of what they do. I'm sure they'd love to understand what we do, and have that kind of mingling, and I know that's not an easy ask. But I think in general, just like the cohesion within the divisions, which I think also stems to the stipend issue. Because there's a lot of

212

00:42:19.450 --> 00:42:34.820

P1.3: uh discussion among the divisions of like their stipend differences, and why they don't want to do like a certain amount. So I feel like that That kind of propagates the uh lack of continuity among among the groups.

213

00:42:39.670 --> 00:42:56.189

P1.2: Yeah, I agree. I I feel the expectations for first and second years is about the classes, and the exam. And the exam is maybe not that good, but the courses and the expectations are good. And after that

214

00:42:56.200 --> 00:43:15.630

P1.2: The committee meetings for qualification exams is basically based on your research progress. And um, I think that part is very well organized. And I also agree that after the second, after the first year of the classes it's hard to meet

215

00:43:15.640 --> 00:43:21.070

P1.2: other people in other divisions. So yeah, if there is more events. Um.

216

00:43:21.280 --> 00:43:23.250

P1.2: maybe it's helpful.

217

00:43:36.030 --> 00:43:43.729

Moderator: Okay? And what other thoughts or suggestions would you like to share? Um, That, you think would be important for this study?

218

00:43:54.890 --> 00:44:05.869

P1.2: Maybe let us know the results. I'm curious about what others are thinking and how this study can have some impact for the school?

219

00:44:10.000 --> 00:44:21.120

Moderator: Yeah, i'm excited to, and really hope that these suggestions that we can take to the Wellness committee will really improve our schools’ overall well being

220

00:44:22.120 --> 00:44:23.729

Moderator: anything else?

221

00:44:28.380 --> 00:44:32.740

P1.3: I feel like, yeah, like everything we discuss is is pretty much

222

00:44:32.750 --> 00:45:01.720

P1.3: what uh I had feelings on like getting out there in terms of like wellness and everything. And um, you know, like I sit on the [graduate student government]. And so it's really nice that we have it. Um, I do wish there was like more we could do for students, I think, in the school of pharmacy, and, you know, cooperating with the PharmDs as well doing, like, you know, across the [school] events as a as a huge division, or as a huge school, would be really nice as well. Um! Just to see that

223

00:45:01.730 --> 00:45:11.019

P1.3: sometimes it feels like we're alone, but it's like we're. We're really like a big community, and we should kind of like, get to know each other in some sort of aspect. So

224

00:45:11.170 --> 00:45:12.080

yeah,

225

00:45:19.190 --> 00:45:21.089

Moderator: P1.1, Any last thoughts?

226

00:45:24.450 --> 00:45:27.870

P1.1: No, I think I think we covered everything that I wanted to mention.

227

00:45:28.640 --> 00:45:30.049

Moderator: Okay, great.

228

00:45:30.060 --> 00:45:56.559

Moderator: Well, thank you again for um participating in this research. Your input is very valuable to our community, and i'm sure the findings for the study will help to inform and support future. Well, being efforts. If you happen to think of anything else you'd like to share. Please use um. The survey link that's included on um the calendar invite for today. Um! And you will be able to anonymously provide any additional feedback should you do so.

229

00:45:56.570 --> 00:46:00.980

Moderator: Um, so thank you again, and I hope you all have a happy Thursday.
