# Supplementary material for: Assessing factors that influence graduate student burnout in health professions education and identifying recommendations to support their well-being
Source: PLoS One. 2025 Apr 15;20(4):e0319857. doi: 10.1371/journal.pone.0319857 (PMC11999156; doi:10.1371/journal.pone.0319857)
Supplement: S3 File — (DOCX) [file pone.0319857.s003.docx]

**S3 File. ABD Focus Group Transcript**

10/12/2022

1

00:04:49.340 --> 00:04:51.620

Moderator: Hello, everyone, How are you guys doing?

2

00:04:53.540 --> 00:04:55.510

Not too bad. How are you doing?

3

00:04:56.180 --> 00:04:59.039

Moderator: Great, Thank you. Can you guys hear me? All right?

4

00:05:01.580 --> 00:05:17.889

Moderator: Okay, Sounds good. Well, um! We'll go ahead and get started. Thank you both for joining me today to discuss Phd: Student: Well, being programs. Um, we greatly appreciate your time and helping us improve our school through the study.

5

00:05:17.900 --> 00:05:30.329

Moderator: My name is [blinded], and I am a pharmacy student who will be conducting the focus scripts today. Um! I work alongside [blinded], and [blinded], as well as [blinded], who is overseeing this project as pi

6

00:05:30.600 --> 00:05:40.389

Moderator: Uh. This project is entitled Identifying Structures. That impact well being Um, our Irb number, if you're interested, is two, one, one, six, two, nine

7

00:05:40.400 --> 00:06:06.440

Moderator: um. We know you received a copy of the informed consent. When signing up this, we will just briefly recap it. Now, um! This focus group interview will be recorded and all data will be de-identified prior to analysis in dissemination uh discussions and comments shared in the focus. Group will be identifiable by other focus. Group participants and participants are reminded not to disclose any specific comments or dialogue with others outside the focus group meeting

8

00:06:06.450 --> 00:06:31.240

Moderator: for the purpose of our discussion Today we we will be focusing on well being and burnout domains. While these domains were evaluated in the march, twenty twenty one well being a baseline assessment in which many of you participated. Um, while the baseline assessment quantified the well being uh the well being assessment. The purpose of this study is to further explore the qualitative factors that contribute to these domains.

9

00:06:31.250 --> 00:06:40.970

Moderator: These domains are defined as burnout, which is characterized by prolonged or repeated periods of stress, where a person begins to feel mentally exhausted by their tasks.

10

00:06:40.980 --> 00:06:59.290

Moderator: and, well being, which is characterized as a state of being happy, healthy, and prosperous.

11

00:06:59.300 --> 00:07:02.349

Moderator: Um, to either. Do you have any questions before we begin.

12

00:07:04.540 --> 00:07:06.509

A2.2: That makes sense.

13

00:07:06.750 --> 00:07:15.540

Moderator: All right. Great. So um! Our first question is what factors positively affect your well-being or bring you fulfillment.

14

00:07:27.140 --> 00:07:47.039

A2.1: Um! Just a couple to get started. Of course I think work life balance being your Phd student is an easy one. Um, because you can really tell when it's not there, and it makes a huge difference when it is there. Um, I think also, having fulfillment that, you know, fulfilled life, be that intellectual fulfillment, or otherwise is really important.

15

00:07:50.510 --> 00:07:52.630

Moderator: Yeah, definitely. Thank you for sharing.

16

00:07:55.010 --> 00:07:57.480

A2.2: Yeah, I agree with that. Um,

17

00:07:57.690 --> 00:08:05.980

A2.2: I think that supportive um like just like supportive people, but also just like supportive environments.

18

00:08:06.180 --> 00:08:14.149

A2.2: Um! contribute a lot to my well being um, and I do think, like A2.1 said, a sense of fulfillment.

19

00:08:14.580 --> 00:08:15.770

A2.2: helps

20

00:08:16.010 --> 00:08:19.390

A2.2: keep me looking forward to things.

21

00:08:19.590 --> 00:08:24.520

A2.2: Um. So feeling like i'm making progress, even if it's

22

00:08:24.560 --> 00:08:30.749

A2.2: small progress, or even if it's progress unrelated to my research, um.

23

00:08:30.830 --> 00:08:32.860

A2.2: The feeling that I have

24

00:08:34.820 --> 00:08:45.289

A2.2: like something I have accomplished, something that makes me feel fulfilled and makes me feel proud helps balance my sense of well being

25

00:08:49.280 --> 00:09:02.220

Moderator: definitely uh, A2.1, I heard you mentioned um work - Life balance is something that positively affects your well being. Um, Do you mind diving into that a little bit more how you feel like you go about attaining work, life balance,

26

00:09:03.800 --> 00:09:10.319

A2.1: I'm: sure. Yeah, I mean to be honest right now it's not really happening. Um, And I think

27

00:09:10.340 --> 00:09:27.679

A2.1: it's a difficult problem to address, because there are factors that are within students control. Um, And so I think we can definitely learn to manage those factors, and I think throughout our training we probably get better at adjusting them.

28

00:09:27.690 --> 00:09:44.709

A2.1: Um, but there's also a lot that's out of your control. When you do research, and when you're trying to get a PhD um. And so you know, depending on the flow of those things that are out of your control, I think sometimes it feels like It's a little bit out of your hands, and it's more how you address the situation.

29

00:09:44.750 --> 00:09:57.149

A2.1: Um, but I would say. It always feels like you're gonna pay for your time somehow or another. So, because we have requirements to graduate, going slower

30

00:09:57.270 --> 00:10:16.480

A2.1: is maybe better for your day to day work, life balance. But then, if you're here for another year or two, Um! That might not be great for your financial well being, and that can be a stressor that will get you on the other side. Um. So in some ways it can feel like a zero sum game. But I think again, just learning

31

00:10:16.490 --> 00:10:31.340

A2.1: how your specific um schedule and workload, which I think is really diverse, depending on even what program we're in, and you know who's lab we’re in, or what kind of research we're doing? How that um is sort of individualized to you.

32

00:10:31.350 --> 00:10:42.060

A2.1: Um As you work in your field. I think hopefully over the years that can improve at least the factors that we can control when it comes to work-life balance.

33

00:10:45.580 --> 00:10:47.200

Moderator: Thank you for sharing

34

00:10:54.420 --> 00:11:02.380

Moderator: Do either of you feel strongly about maybe how the workplace affects your um well, being positively, or maybe any work relationships, you may have um or school relationships?

35

00:11:07.530 --> 00:11:09.530

A2.2: Can you repeat the question?

36

00:11:09.560 --> 00:11:20.759

Moderator: Yeah, of course, um do. Either of you feel um strongly about any, maybe workplace or school place relationships that you feel contribute to your well being or bring you fulfill it.

37

00:11:26.730 --> 00:11:46.649

A2.2: I think there are two types of workplace relationships that contribute to my sense of well being, and one is my relationship with faculty members, my adviser. Um. I guess people that I would consider to be my mentors are the leaders of my workplace environment.

38

00:11:46.660 --> 00:12:04.089

A2.2: So, feeling like I receive support from my advisor that she acknowledges my need for well being and helps me work toward a work life balance that works for me is very important, and it's nice when that

39

00:12:04.100 --> 00:12:07.700

A2.2: sentiment is reflected not only in

40

00:12:08.080 --> 00:12:19.400

A2.2: the relationship with my adviser, but with the other faculty and the other leaders in my division. So I know It's not just one person that cares about this, but um

41

00:12:20.070 --> 00:12:23.719

A2.2: multiple people that it's sort of like a collective effort.

42

00:12:23.750 --> 00:12:38.900

A2.2: Um! And then I think my relationships with the other students in my division. Um, but it cross. I think the PhD Program more broadly. Those are important for establishing a sense of well-being, because

43

00:12:39.220 --> 00:12:43.809

A2.2: the other Phd students are the ones that have

44

00:12:43.970 --> 00:12:54.400

A2.2: the best idea of what I'm going through. So if I feel overwhelmed by my research or my classwork, or

45

00:12:56.040 --> 00:13:08.220

A2.2: even trying to balance my professional life in my personal life. My peers are the people that I feel like, understand that most because they are likely experiencing the same

46

00:13:08.300 --> 00:13:13.960

A2.2: like stressors when it comes to class work and similar issues when it comes to research.

47

00:13:14.070 --> 00:13:16.370

A2.2: Um, And so they can,

48

00:13:17.070 --> 00:13:20.950

A2.2: or like. We can empathize with each other and share

49

00:13:21.500 --> 00:13:22.569

A2.2: sort of

50

00:13:23.240 --> 00:13:25.290

A2.2: emotional and like

51

00:13:25.710 --> 00:13:28.710

A2.2: mental support for

52

00:13:29.670 --> 00:13:34.040

A2.2: overcoming challenges that we face as PhD Students.

53

00:13:41.020 --> 00:14:06.499

A2.1: Um, I definitely support everything that A2.2. Just said Um, Sorry also just realized. Maybe I shouldn't use names. I don't know if that makes your job harder.

Moderator: But you're good, you can.

A2.1: So I I definitely confer that there is two main kind of roles that people that are on campus tend to play in our lives, because, you know, they're either mentors or students typical. There's not many other people that we interact with frequently.

54

00:14:06.680 --> 00:14:08.030

A2.1: Um.

55

00:14:08.290 --> 00:14:22.989

A2.1: And I do think that the converse is true, and I want to highlight that as well. So I think, having mentors, PIs, other faculty or members of our community that have um some

56

00:14:23.130 --> 00:14:36.420

A2.1: hierarchical role over us that are not supportive of work like the balance, or who lack to address it at all. Um! That can of course be incredibly negative.

57

00:14:36.430 --> 00:14:46.820

A2.1: Um. And so I think I often see this enter play between the students supporting each other and trying to help each other find

58

00:14:46.940 --> 00:15:05.770

A2.1: um something sustainable. Um! And then kind of a pushing pull from mentors depending on your specific situation. Um, So I think. Well, I've been here. I've had kind of a range of experiences with mentors um and their ideologies.

59

00:15:05.780 --> 00:15:20.510

A2.1: Um, and Sometimes, too, it isn't as much their view of work like balance, or their view of wellness, but more of their ability to support you in your research, because if you're not getting benefit and support

60

00:15:20.520 --> 00:15:31.890

A2.1: um, then that is gonna take its toll and regardless. If your boss is fine with you, taking your vacation or not. You're still going to struggle, and that's going to affect your wellness um significantly.

61

00:15:39.150 --> 00:15:51.579

Moderator: Yes, definitely. Thank you both for sharing all those different um viewpoints and perspectives. I think that I definitely hear community coming out as a theme and um support from Mentors and others around you.

62

00:15:51.590 --> 00:16:08.579

Moderator: Um, and I know you both early, both mentioned earlier feeling um the sense of fulfillment, and just doing what in what you're doing matters um do both of you also feel like not only feeling fulfilled in your work, but also feel like also feeling like

63

00:16:08.590 --> 00:16:15.520

Moderator: your work is valued by others. Um! Is that important in um? Your sense of well being at all.

64

00:16:23.340 --> 00:16:39.899

A2.1: Oh, I actually feel like, since being in this program, my relationship to others, perceptions of me and my research has really shifted. So I think, before I came here I was incredibly motivated by the assessment of others on my research.

65

00:16:39.910 --> 00:16:51.689

A2.1: Um, and I think I've really been able to hone since coming here. Um! An awareness that not everyone has all of the information. So sometimes

66

00:16:51.730 --> 00:17:10.629

A2.1: as a Phd and then my experience not sure if this relates to A2.2 or others. But um, you get really positive feedback for positive results, and so negative results might have been just as hard to obtain, and might be meaningful as well, and might showcase your skills whatever um,

67

00:17:10.790 --> 00:17:12.710

A2.1: and to not get

68

00:17:12.839 --> 00:17:15.750

A2.1: uh the same level of

69

00:17:16.000 --> 00:17:31.579

A2.1: I don't know appreciation for those results. I think matters a lot less to me now um than it used to so well. I do think it is definitely important to feel like the community is aware of what students are doing, and I love,

70

00:17:31.590 --> 00:17:44.009

A2.1: you know, learning about what other students are doing and helping them to see how awesome it is. I also think that um to some extent being able to discern the degree to which that um

71

00:17:44.520 --> 00:18:01.920

A2.1: I don't. I just want to say appreciation, but that I Don't know Um. Sentiment is like. Is it really valuable to your experience? Is it really reflective of the work that was put in? the quality of the research? or the impact of um your work? I guess.

72

00:18:17.620 --> 00:18:26.289

A2.2: I think I thought about this question a little differently. I do think it’s helpful to your sense of well-being to have other people think your work is valuable, but I don't think it's.

73

00:18:26.420 --> 00:18:28.879

A2.2: I don't think it's necessarily

74

00:18:28.970 --> 00:18:33.380

A2.2: the biggest driver of well-being, because

75

00:18:34.140 --> 00:18:52.670

A2.2: and and this might be very clouded by my experience as a graduate student, but I work with um an advisor at PI, who is still a very new faculty member, and so a lot of the projects that we do they're kind of small. She's still sort of building up her lab.

76

00:18:52.690 --> 00:19:01.500

A2.2: She's not working on like millions of dollars or a one funding it's not huge projects. And so the the projects that I work on

77

00:19:01.540 --> 00:19:02.720

A2.2: our

78

00:19:02.740 --> 00:19:04.449

A2.2: going on to

79

00:19:04.480 --> 00:19:11.890

A2.2: be published in like huge journals, or anything like that, and i'm i'm aware of that. And so it's not a place that

80

00:19:12.560 --> 00:19:31.119

A2.2: I seek like a sense of fulfillment from. I think I have a a very, I think I've very much scaled my expectations, and so I know that my work is valuable to my adviser, and I know that people in my division find my work to be interesting, and they find it to be valuable.

81

00:19:31.130 --> 00:19:50.269

A2.2: But, um! The The impact factor of my work is still small, and in some ways I think that is maybe applicable to all graduate students because we're all just working on our own single projects, and we're still trainees. We're not.

82

00:19:50.280 --> 00:19:53.330

A2.2: We're not like great minds

83

00:19:53.970 --> 00:19:59.140

A2.2: creating waves in our fields at this point. Um! And so

84

00:19:59.600 --> 00:20:05.090

A2.2: I I guess I It's just not feedback that I

85

00:20:05.170 --> 00:20:24.659

A2.2: would necessarily expect, and so it's not something that I rely very heavily on, I think, just as like a very micro scale. It's nice to know that my immediate peers, like the other members of my lab and my adviser, and and, like the people immediately around me, think my work is interesting.

86

00:20:24.670 --> 00:20:40.330

A2.2: Um, but it's not impactful to my well being on a large scale, I would say Um, but I I want to clarify that with I think people in other labs might have different experiences, and so it will probably very

87

00:20:40.360 --> 00:20:44.719

A2.2: um by graduate student by the type of research that you do. Um,

88

00:20:48.240 --> 00:20:55.100

Moderator: Thank you both for sharing those great perspectives. Um, it's always interesting to hear um how people kind of take that in

89

00:20:55.430 --> 00:21:01.320

Moderator: um. Does anyone have any other thoughts on factors that positively affect Well, being

90

00:21:07.160 --> 00:21:17.679

Moderator: all right, hearing none, i'm going to move on to our next question. Um, which is what factors negatively affect your well-being, and or cause burnout

91

00:21:34.050 --> 00:21:48.209

A2.1: um I guess some easy ones to name, of course, poor work like balance. Um, not feeling like you have um a trajectory not having a good structure to your training. Um,

92

00:21:48.330 --> 00:21:56.540

A2.1: I think. Of course, mentorship, you know. Sorry I like I should probably say the ones that are obvious, but I I do think um

93

00:21:58.530 --> 00:22:03.380

A2.1: sorry. Let me think of like how to explain this. I think that

94

00:22:03.470 --> 00:22:18.620

A2.1: when you work in a field like research that's so open ended Um, having structure not just been like, Okay, I want to have this done by whenever, because obviously those goals don't always come to fruition, but having structure in terms of

95

00:22:18.630 --> 00:22:36.819

A2.1: um concrete training goals that Aren't tied to the outcomes of your research, but rather the skills that you require. I think, having those is incredibly helpful. And I think most of our programs don't, have those, you know I get i'm sure it totally depends on your PI and your training plan on your mentors are

96

00:22:36.830 --> 00:22:56.420

A2.1: um, but because our fields are often tied around benchmarks of publication and of significance of research rather than significance of training. I think it is difficult to sometimes see the gains that you're making um, and only focus on the path that your research is taking. And so I think

97

00:22:56.430 --> 00:23:13.400

A2.1: what I've seen of students who are experiencing burnout is that that significantly affect their ability to feel like they're making a difference. They're moving forward. And of course, when you feel like your work is for nothing, it's really difficult to keep working at the pace that's required. Um of a program like this.

98

00:23:22.170 --> 00:23:23.899

A2.2: I think that

99

00:23:25.290 --> 00:23:28.500

A2.2: at like a sense of uncertainty in

100

00:23:28.650 --> 00:23:30.289

A2.2: your environment

101

00:23:31.370 --> 00:23:32.849

A2.2: really impacts

102

00:23:32.890 --> 00:23:35.680

A2.2: really negatively impacts my

103

00:23:36.060 --> 00:23:44.049

A2.2: well being, and I think that sense of uncertainty can come from a lot of different sources like A2.1 has mentioned sort of like

104

00:23:44.260 --> 00:23:59.730

A2.2: feeling uncertain about if you're making progress in your research, and uh feeling uncertain about the expectations that your division or your adviser have, for you can be a big sense of uncertainty.

105

00:23:59.750 --> 00:24:01.509

A2.2: In some ways

106

00:24:01.890 --> 00:24:21.620

A2.2: graduate students have to accept like a level of uncertainty, because research is, is about asking questions that Don't have clear answers, and that's the point of what we do. Um, But because you're kind of dealing with that uncertainty you're confronting new challenges in your research on sometimes like,

107

00:24:21.770 --> 00:24:27.169

A2.2: and sometimes that happens every day that there is a new uncertainty that you have to deal with.

108

00:24:27.660 --> 00:24:28.640

A2.2: It's.

109

00:24:30.090 --> 00:24:40.539

A2.2: It reaches a level of being overwhelming when uncertainties by up in other areas. So I think sometimes about

110

00:24:41.780 --> 00:25:00.799

A2.2: like my Phd program having two signs and one side is like the research side and one side is is like the academic institutional side, where I take classes, and I fill out forms, and I and I go through the the steps

111

00:25:00.810 --> 00:25:20.160

A2.2: of getting a formal degree from the University, and sometimes that side creates a lot of uncertainties where I have to take these classes, but i'm not sure which ones to take, because they keep changing the list and the websites never up to date. And I don't know what's going on, so I can't make

112

00:25:20.170 --> 00:25:22.420

A2.2: decisions about

113

00:25:22.900 --> 00:25:27.199

A2.2: where I should be going, and then I feel uncertain, and so,

114

00:25:27.360 --> 00:25:33.069

A2.2: making sure that there is certainty in the rest of the environment, so that

115

00:25:33.130 --> 00:25:50.790

A2.2: it doesn't feel like you're just lost where you don't know what's happening in your academic like life, and you don't know what's happening in your research is just overwhelming uncertainty, and that really negatively affects my well being so, even though you can't remove all uncertainties,

116

00:25:50.800 --> 00:26:04.939

A2.2: making things as clear as possible, and providing structure where where it can be, I think would be, would be helpful. Um, preventing sort of unnecessary uncertainties in your environment

117

00:26:05.720 --> 00:26:06.860

A2.2: would help

118

00:26:07.400 --> 00:26:09.990

A2.2: in increase. Well, being, I think

119

00:26:14.520 --> 00:26:28.230

A2.1: I really agree with that so strongly I was. I don't know everything you just said. I feel like ties really well into resiliency as well. We're adding a maybe just my experience. But I feel like most people have

120

00:26:28.320 --> 00:26:46.399

A2.1: a capacity for dealing with uncertainty and change um and people's ability to be flexible, I think, can be increase with things that increase our resiliency. But everybody's got a cap, and I think I would guess, for most Phd. Students are

121

00:26:46.410 --> 00:27:01.390

A2.1: sort of flexibility. I don't know. Bank account is already almost spent just from dealing with the variability that we um have to address with our thesis work. And so there's so little room um to deal with other

122

00:27:01.400 --> 00:27:15.069

A2.1: uncertainties, other changes in our environment that significantly affect us, because so much of our existence is driven already by uncertainties that we have no ability to address. We kind of just have to take them as they come.

123

00:27:15.080 --> 00:27:21.870

A2.1: Um! So just wanna like Super agree with that. I think most Phds that I interact with

124

00:27:21.910 --> 00:27:33.069

A2.1: feel that way as well, and I can tell the closer people get to burn out. And same for my own experience, the worse people become at handling um.

125

00:27:33.270 --> 00:27:50.729

A2.1: Change your unexpected things in our environment, and typically, or at least with me, that's usually sort of gone hand in hand with an increase in variability or unexpected things with research. So just yes, like any way to mitigate uncertainty or unnecessary

126

00:27:50.740 --> 00:27:54.139

A2.1: stress would be so great,

127

00:28:02.670 --> 00:28:23.949

Moderator: I think. Um, you both bring up really um interesting point about the Phd program in this is that you are really dealing with two separate worlds of research and the academic curriculum. Um. Are there any other um factors about like that balance between the research and your curriculum stressors that may negatively affect your well being your cause. Burnout

128

00:28:31.360 --> 00:28:48.340

A2.1: um, maybe more on the positive side. So I know two of us are in different divisions, and I know my division has some of the fewest classes, some of the fewest requirements. Um! And that was a huge driver for me actually coming to this program, and I really appreciated it.

129

00:28:48.350 --> 00:29:05.760

A2.1: Um! Which is not to say that classes and the academic side isn't valuable, but I do think that um the approach that was taken which is very much like a student driven um way to fulfill your requirements, so that you can tailor your

130

00:29:05.770 --> 00:29:24.779

A2.1: classes to your own thesis needs, and you also have a ton of flexibility about when you take classes, and the requirements are really low, so you can overload. If you feel like you need them. But if you don't, you don't have to. I think that really has proved the quality of life of most of the students in my division, because I know it. Didn't always used to be like that.

131

00:29:24.790 --> 00:29:36.799

A2.1: Um! And again, every division has really different, you know, science and like uh, I guess perspectives within their division. So I don't know if that would necessarily help or not. Um,

132

00:29:36.990 --> 00:29:50.090

A2.1: but also, I do think just um minimizing the hoops that we have to jump through outside of classes. Um! But I think all the divisions have a qualifying exam that's multi-part um, And so

133

00:29:50.100 --> 00:30:07.980

A2.1: I felt like my divisions is really kind of cut and paste. It was really straightforward, and I appreciated that as well. Um, but I know not. Every program is like that, and having uncertainty to your confusion about what requirements needs to be met, um can definitely be a negative contributor.

134

00:30:17.870 --> 00:30:34.770

A2.2: I think that’s a great point A2.1. I think that the qualifying and comprehensive exams that we take as Phd. Students. Those are a source of incredible stress for students, and you know that they're coming from the like. The Your first day as a Phd. Student like you know that exam is coming up,

135

00:30:34.820 --> 00:30:39.530

A2.2: and um! You kind of hear about it from other students,

136

00:30:40.050 --> 00:30:46.239

A2.2: and sometimes it seems like it's a black box of you don't know what you're you're getting into. But, like

137

00:30:46.280 --> 00:30:50.389

A2.2: you're on one side of it, and the older students are on the other side of it.

138

00:30:50.520 --> 00:30:51.770

A2.2: Um,

139

00:30:51.910 --> 00:30:56.739

A2.2: there's I think, a lot of stress and uncertainty around

140

00:30:56.910 --> 00:31:06.399

A2.2: comprehensive exams for younger students in Phd Programs, and I think A2.1 and I both have the benefit of being

141

00:31:06.950 --> 00:31:14.240

A2.2: oh, like past those exams right now, so I can like, Look back and and think about that. But

142

00:31:16.270 --> 00:31:28.360

A2.2: i'm not at all advocating that, like those exams shouldn't exist, but acknowledging that they are a source of stress for students, I think, would go a very long way um

143

00:31:28.660 --> 00:31:36.530

A2.2: towards improving well, being just by like making requirements clear, making it

144

00:31:37.240 --> 00:31:41.720

A2.2: less of a mystery for students. Um,

145

00:31:42.240 --> 00:31:54.589

A2.2: there's so many other things that you're trying to already balance like you're already trying to balance from my in. In the case of my division we have to take two whole years of classes, which I think is one of the longer um

146

00:31:54.710 --> 00:32:12.130

A2.2: that amounts, of course, requirements. So you're already trying to balance that you're trying to balance your your research. You're trying to find a dissertation topic. There's already so many other things going on, and and that one could could be lessened a lot. Um, the stress that comes from this sort of exams and

147

00:32:12.140 --> 00:32:15.430

A2.2: like um requirements for candidacy

148

00:32:15.550 --> 00:32:20.749

A2.2: through better communication. Um, through just more structure in that

149

00:32:20.880 --> 00:32:24.140

A2.2: process, I think, would help students a lot.

150

00:32:31.230 --> 00:32:49.659

Moderator: Thank you both for sharing this thoughts. Um, I know we mentioned, or you both mentioned earlier. Um! When we were talking about factors that positively affect your um. Well being was the relationship with um. Not only your community and the students around you, but also a good relationship with your mentors and um.

151

00:32:49.670 --> 00:33:07.650

Moderator: People in your committees. Um. Are there any experiences that you may have had, and not specific experiences, But just um talking about it in the sense of the word um with supervisors or um mentors that have that can negatively affect your well being um or cause burnout

152

00:33:10.070 --> 00:33:27.109

A2.1: one hundred percent. Yes, uh, I just want to clarify. So would you like us to give examples like general examples, or just like you guess or no sort of a situation here?

Moderator: Um examples. We're fine. You'd be like i'll be de-identified. So um please feel free to take your mind.

153

00:33:27.120 --> 00:33:42.349

A2.1: Got you? Yes, okay. So for sure one hundred percent um. And the experience that i'm about to share has also affected other students. So this is something also worth mentioning that at first, like one on one interactions nearly affecting, is, but also

154

00:33:42.520 --> 00:33:57.250

A2.1: environments that are set by mentors can affect all the students, and that is really bad. Um: So yeah, I've had experiences with mentors. Um, who will

155

00:33:57.540 --> 00:34:15.679

A2.1: create personal situations that Don't need to be personal. Um. So I think professionalism, of course, is really important in any context, but especially in a high stress field like the ones that we work in.

156

00:34:15.690 --> 00:34:17.830

A2.1: Um: So

157

00:34:18.380 --> 00:34:33.430

A2.1: yeah, I mean, i'm trying to like, put this lightly. But basically I've um had experiences with mentors who will make mistakes, and we all make mistakes, and that's totally fine, like no one's infallible, but then react to their mistakes

158

00:34:33.440 --> 00:34:43.799

A2.1: by blaming students, regardless of evidence for and to the point where all the students are like. Oh, my gosh! Like i'm so confused like, was that me? How could that happen, and so

159

00:34:43.989 --> 00:34:53.700

A2.1: I want to use the term gaslighting, and I don't know if this is like a conscious effort. Um! But I have encountered

160

00:34:53.790 --> 00:35:12.119

A2.1: specific individual who has this behavior pretty constantly, and it has dramatically decreased the quality of life of all of the students who work with this Um Mentor um, and it just like repeated behaviors. So

161

00:35:12.440 --> 00:35:31.530

A2.1: I guess really quickly. I don't like take all the time on this one but um! Another kind of interaction that I think negatively affects students well. Being um is a lack of acknowledgment of situations like that existing um. So this was brought to another Mentor's attention

162

00:35:31.540 --> 00:35:51.470

A2.1: and immediately shot down despite multiple students, independently having this experience, and so that you know, I think there's like, What do you do when there's an unfortunate interaction, And Then what do you do when that interaction is brought up in the dismiss? And I think that

163

00:35:51.480 --> 00:36:02.660

A2.1: really amplified the negativity surrounding that experience for all of the students. Because again, you feel like you're not being taken seriously. Your experiences aren’t valued,

164

00:36:02.720 --> 00:36:14.699

A2.1: and regardless of the reason why that was the reaction. Um, I think the quality of life and the quality of work that students in this environment, including myself, has been able to

165

00:36:14.870 --> 00:36:20.060

A2.1: provide, was definitely impacted by that situation.

166

00:36:31.280 --> 00:36:32.470

A2.2: Um,

167

00:36:34.870 --> 00:36:45.119

A2.2: I think. Um, thank you for sharing that, A2.1. Um. I haven't had such similar experiences, but I I think

168

00:36:46.310 --> 00:36:47.379

A2.2: that

169

00:36:47.720 --> 00:36:57.129

A2.2: a sort of like level of openness is really valuable between Phd students and their their advisors and their mentors,

170

00:36:57.440 --> 00:37:01.879

A2.2: and that can contribute to well being. But when that's not

171

00:37:02.380 --> 00:37:05.670

A2.2: present, I think that really does take away from

172

00:37:05.750 --> 00:37:07.219

A2.2: well being,

173

00:37:07.780 --> 00:37:13.590

A2.2: because it it means that Phd. Students and and their advisors and their faculty,

174

00:37:13.740 --> 00:37:28.659

A2.2: aren't on the same page about what they're experiencing, and so then they can't make the best decisions. And and sometimes I I feel like we have an issue or not not. We like i'm not pinpointing anyone, but I think

175

00:37:28.850 --> 00:37:44.189

A2.2: in general I think we have an issue where we think that we've established an open communication, and, like we think that we have this openness, but we really don't, and sometimes I I think I think back to like

176

00:37:44.500 --> 00:37:53.469

A2.2: a conversation I had with my professor once. It was maybe a at like the beginning of a class, or it was at the beginning of like a

177

00:37:53.510 --> 00:37:56.119

A2.2: a zoom meeting, and

178

00:37:56.240 --> 00:37:57.339

A2.2: they

179

00:37:57.400 --> 00:38:05.770

A2.2: they just like posed a question to the group. They like, How's everybody doing? And I feel like they were shocked when people were like we're a little

180

00:38:05.780 --> 00:38:18.869

A2.2: a little bit stressed. It's the end of the semester. We all have like four papers and two projects and research that we're trying to do, and they seem like very surprised that people answered that question honestly,

181

00:38:18.880 --> 00:38:34.350

A2.2: and people were like Oh, no, I'm i'm feeling a bit stressed and a bit overwhelmed right now. Um. And so in that case I feel like that was an opportunity to have like an an openness between the the students and the faculty, and

182

00:38:34.360 --> 00:38:52.900

A2.2: and to get everyone on the same page. Um! That wasn't, in fact, a real opportunity like that person was surprised when when there was that level of openness and honesty. Um! And so when that's not an expectation. And and when that doesn't exist,

183

00:38:53.740 --> 00:38:58.430

A2.2: I think that takes away from from our well being, because

184

00:38:58.580 --> 00:39:08.340

A2.2: to acknowledge that there is an issue with your well being. That you're struggling with something is a very meaningful and important part of

185

00:39:08.950 --> 00:39:13.539

A2.2: creating the change that you need to ensure your well being um,

186

00:39:13.560 --> 00:39:25.120

A2.2: And I think that that happens where we we like, ask people how they're doing. And then but we don't really expect them to share, and we we do these things to do them, but not

187

00:39:26.270 --> 00:39:29.040

A2.2: we don't have the real intention of

188

00:39:29.690 --> 00:39:33.860

A2.2: like creating the openness that needs to exist.

189

00:39:38.270 --> 00:39:57.750

A2.1: Is it Okay, If I add on that a little, because very much relate to that as well. Um. And I feel like I've encountered that issue in multiple areas of our community. Um. So while we do have a really, I think, unique, awesome, supportive community here. Um,

190

00:39:57.760 --> 00:40:13.830

A2.1: It's also easy to forget that It's okay, and not be okay. You know that's really like uh, I don't know. Passe to say. But um, I often do find situations like that occurring with mentors um or with

191

00:40:14.090 --> 00:40:16.419

A2.1: um like [School] leadership

192

00:40:16.450 --> 00:40:31.320

A2.1: where there's so much positivity surrounding what we do well, and not enough acknowledgment of what could be improved almost to the degree where it feels like. No one's willing to kind of check under the hood, and really see how things are going.

193

00:40:31.330 --> 00:40:51.060

A2.1: Um! And when conversations about things that should be improved which can be scary, but really need to happen, are shut down or quickly reframed to only focus on the positives and neglect things that really need addressing. I think that also contributes directly to burnout, because I know that

194

00:40:51.070 --> 00:40:57.530

A2.1: it's common among um more senior students, at least in my program, to feel um

195

00:40:57.560 --> 00:41:08.179

A2.1: incredibly jaded about the program and feel like there's a lot of lip service, but not a lot of actual intent to address issues as they arise.

196

00:41:08.190 --> 00:41:24.179

A2.1: Um! And so again, feeling like there's no one really in your corner, or that your experience is not going to be validated or real change will never happen, regardless of the effort that you put in to the system, I think, has really negatively affected.

197

00:41:24.290 --> 00:41:27.040

A2.1: Um. A number of students,

198

00:41:27.230 --> 00:41:29.339

A2.1: including myself at times.

199

00:41:35.290 --> 00:41:46.020

Moderator: Thank you both for sharing that um, And it's definitely coming from a place of openness and little bit vulnerability. And I think it's definitely something important that needs to be talked about. So I really appreciate that.

200

00:41:46.150 --> 00:41:57.280

Moderator: Um! Are there any other thoughts that come to mind when you think about things that negatively affect your well being, or contribute to burnout before we move on to the next question. Just want to make sure we're touching it all.

201

00:42:06.310 --> 00:42:21.149

Moderator: Okay. So our last question. And um, both of you have have shared a couple of these already. Um. Which I really appreciate. But our last question is, Um, what recommendations would you suggest to improve graduate student? Well, being at the school,

202

00:42:31.510 --> 00:42:38.220

Moderator: I know. So far we've heard a little bit more structure, and maybe reducing variability and things that can be controlled.

203

00:42:38.300 --> 00:42:39.459

Moderator: Um!

204

00:42:39.500 --> 00:42:44.659

Moderator: To be reducing the stress around qualifying exams and improving communication.

205

00:42:46.770 --> 00:42:49.899

Moderator: Would you like to expand on any of those, or maybe add some more

206

00:42:51.380 --> 00:43:09.990

A2.1: uh, maybe for structure, and this just my opinion so like just one person. But I do want to clarify that structure, not necessarily meeting requirements, because I think a lot of times when we think about academia, it's really easy to think. Oh, it's a structured program. So there's a lot of boxes we need to check. So you know exactly what you need to leave.

207

00:43:10.000 --> 00:43:23.759

A2.1: Um, And I think at least when I think of structure in a Phd program. I think more of like consistency between mentorship experiences between faculty.

208

00:43:23.770 --> 00:43:36.479

A2.1: Um, having more transparency in your relationship with your faculty, mentors, because there really aren’t tools provided to us, at least in my division, to establish um

209

00:43:36.490 --> 00:43:47.280

A2.1: like what the division of labor is going to be, what your expectations are, what your mentors are. It's really up to the Mentor to kind of set the tone. And so students get a really wide variety of experiences here

210

00:43:47.290 --> 00:44:04.309

A2.1: depending on um their lab environment. So I would say more structure in terms of uniformity of experience, but definitely want to make sure. This is not like. We need more things to check off. We need to know exactly what classes and not have flexibility. Um! But

211

00:44:04.320 --> 00:44:08.569

A2.1: other people feel free to disagree like I just just what I would say about that.

212

00:44:10.660 --> 00:44:16.159

A2.2: No, I really agree with that, A2.1. I I feel like you described that really Well, that

213

00:44:16.450 --> 00:44:34.450

A2.2: structure isn't about like more boxes to check, but just being clear about the existing set of boxes that I need to check. Sometimes I feel like there's a list of requirements, and someone somewhere has that list. But I don't have the list, even though i'm the one supposed to be meeting those requirements.

214

00:44:34.460 --> 00:44:45.200

A2.2: So that's the sort of structure it's not making a list longer. It's really just making it more clear what's already there. Um! Sometimes I think that

215

00:44:45.320 --> 00:44:46.970

A2.2: Academia

216

00:44:47.230 --> 00:45:07.130

A2.2: has like, Like academics like to be busy. We always like to have things that we're doing, and and for researchers a lot of time that's just making sure. The research projects are working on our are moving forward. Um! And for the younger students or the younger PhD Students who are taking classes.

217

00:45:07.140 --> 00:45:08.060

A2.2: Um!

218

00:45:08.910 --> 00:45:15.860

A2.2: The like course, work and exams contribute to that sense of busyness. And then there's always just

219

00:45:16.130 --> 00:45:23.789

A2.2: extra stuff that that comes up. I feel like I get so many emails um from the School of pharmacy about

220

00:45:24.250 --> 00:45:38.939

A2.2: different opportunities like today. I feel like I got like five emails about fellowship opportunities that I now need to read through and consider. And there's there's always so many opportunities to be busy,

221

00:45:39.310 --> 00:45:41.509

A2.2: and I think

222

00:45:42.430 --> 00:45:46.489

A2.2: I don't want to say reduce that because

223

00:45:47.090 --> 00:45:50.170

A2.2: opportunity is good. But

224

00:45:50.320 --> 00:45:54.189

A2.2: I sometimes feel like when you're when so many,

225

00:45:55.040 --> 00:45:57.189

A2.2: so many options are like

226

00:45:58.000 --> 00:46:00.270

A2.2: with like thrown your way.

227

00:46:00.370 --> 00:46:19.210

A2.2: I I feel like I I sometimes blindly accept things like Yes, I will do this now to this, and I I don't take the time to think like critically about what I should be doing and what's the most valuable way to spend my time, and sometimes I and I think that that could be

228

00:46:19.490 --> 00:46:22.380

A2.2: a a more helpful mindset; that,

229

00:46:22.440 --> 00:46:28.160

A2.2: as like an institution, I think that we need to adapt that we shouldn't just

230

00:46:28.620 --> 00:46:29.750

A2.2: the

231

00:46:29.780 --> 00:46:40.939

A2.2: and then like holding seminars and holding events and pursuing projects just to do that. But we should think really critically about what's most valuable to us

232

00:46:41.440 --> 00:46:45.739

A2.2: and focus on those things. I think we might find that

233

00:46:49.190 --> 00:47:09.100

A2.2: we could have a a little bit of a less chaotic environment. If we did that if we, if we tried to focus more on on just a few things that were, we're really important. Um, and didn't try to to to do so. Such a variety of things. And this is just my opinion.

234

00:47:09.110 --> 00:47:20.020

A2.2: Um, obviously in this school is trying to provide opportunities to people with a very wide variety of interests like there are. There's a whole class of PharmD students

235

00:47:20.110 --> 00:47:23.319

A2.2: There's five different divisions.

236

00:47:24.070 --> 00:47:34.160

A2.2: There's PhD Students who do vastly different types of research, and the school provides opportunities for them all, And that's really wonderful. Um! But

237

00:47:36.040 --> 00:47:44.310

A2.2: that environment can't reach the point of chaos, or it becomes overwhelming for graduate students. And so

238

00:47:44.350 --> 00:47:56.840

A2.2: that's not like a specific program that could be implemented. So I realized i'm not providing you with like a very concrete um solution or intervention to improve well being, but

239

00:47:57.410 --> 00:47:59.970

A2.2: bringing a sort of more

240

00:48:00.280 --> 00:48:03.559

A2.2: focused and like a more thoughtful mindset

241

00:48:03.960 --> 00:48:17.760

A2.2: to the school culture, I think, would be valuable to creating an environment that is more able to prioritize well being and more able to

242

00:48:19.830 --> 00:48:26.490

A2.2: to be an environment that isn't a place where burnout can cultivate this easily.

243

00:48:37.690 --> 00:48:53.120

A2.1: I guess one other thing that might help, and I don't know if this is feasible. Um, which I guess you know, in a perfect world, of course things would be different. Um! But I do think that there is, you know, currently, there's like absolutely no unbiased

244

00:48:53.130 --> 00:49:09.970

A2.1: outlet that Phd. Students can access to communicate about their well being. Um, and there should be, I know, like we have technically like an Hr Department, but I think they're like they do a lot of other things, and that's not their primary

245

00:49:10.310 --> 00:49:13.040

A2.1: concern. Totally understandable.

246

00:49:13.050 --> 00:49:33.499

A2.1: Um! But other than that, it's like, Who are you gonna check in with. Your mentors have invested interest in your productivity. So they're always going to be biased, even if you have a really good experience or a good relationship with them. Other students um well super great to like, be able to communicate event to them. They're not in a position

247

00:49:33.510 --> 00:49:49.939

A2.1: to act on your behalf if something serious happens. So I think just some sort of like one person wants to hear one questioner of like, How are you doing? And you just know that wherever it's going,

248

00:49:49.950 --> 00:50:06.189

A2.1: it's going to someone who both doesn't have invested interest in your productivity in the program, and also someone who's actually in a position to make a change on your behalf of it, needs to be made so to communicate with your advisor, or you know, to I don't know, like

249

00:50:06.200 --> 00:50:16.520

A2.1: I think a lot more brainstorming would need to happen to make sure that that kind of a system would actually be effective. And you know. Positive thing to have.

250

00:50:16.610 --> 00:50:35.770

A2.1: Um, but it's really hard when there is nothing um, and I know there's like there are small things that are being done. But right now it feels like It's more of like a probing of our community to kind of see where everybody is at, rather than like a actual protective mechanism for students to make sure that those who might be struggling or going through a harder time

251

00:50:35.780 --> 00:50:41.519

A2.1: um can access resources, and there's some sort of awareness that that is happening.

252

00:50:46.540 --> 00:51:02.140

A2.2: I think that's a really good point, and I I it. It reminds me that what I think about well being I really think that it's something that it has to be cultivated at so many levels within the school for it to be really impactful to graduate students

253

00:51:02.150 --> 00:51:06.720

A2.2: like we're kind of down here at the very lowest level. Um,

254

00:51:07.010 --> 00:51:15.829

A2.2: Nobody reports to us like we report to our advisors who report to our chairs to report to the Dean, and everyone has.

255

00:51:15.920 --> 00:51:35.220

A2.2: There's There's just so many levels above us. And so, even if graduate students personally make commitment to prioritize their well being, that's not enough to really affect change. There has to be like a commitment on the part of the advisor, and on a on the part of the chair and and going up.

256

00:51:35.350 --> 00:51:39.419

A2.2: I think A2.1's um comment, that

257

00:51:40.860 --> 00:51:53.660

A2.2: if someone is is struggling, it would be helpful to have a resource who wasn't in that hierarchy, and and who could be a sort of unbiased

258

00:51:53.810 --> 00:51:57.090

A2.2: resource to students? Um,

259

00:51:58.370 --> 00:52:13.440

A2.2: I think positive change for well being can flow through that those different levels. But I also think that's where part of our problem comes from is that the stress uh, and like the pressure that is on

260

00:52:13.450 --> 00:52:24.510

A2.2: graduate students as researchers on PI*s* as the leaders of research, and on [university] as a research institution, those the negative stress and negative pressure. They also flow through those

261

00:52:24.580 --> 00:52:27.740

A2.2: and so multi-level

262

00:52:27.770 --> 00:52:32.969

A2.2: solutions or multi-level interventions, or even like A2.1, has post like

263

00:52:32.990 --> 00:52:37.900

A2.2: an intervention. That's outside of those levels, is what will be needed to make

264

00:52:38.670 --> 00:52:44.370

A2.2: improvements in Phd. Student well being, and to make sustainable improvement.

265

00:52:44.590 --> 00:52:56.699

A2.2: Um! I can certainly make a commitment to myself that i'll prioritize well being, but that really does nothing for the graduate students that come after me. Um, that it that's not

266

00:52:57.340 --> 00:53:01.220

A2.2: like that. One level change is not a sustainable um

267

00:53:01.670 --> 00:53:03.240

A2.2: solution to

268

00:53:03.310 --> 00:53:07.300

A2.2: well being and and like mental health and graduate students as a whole.

269

00:53:20.770 --> 00:53:23.689

Moderator: Do you have any other recommendations?

270

00:53:24.260 --> 00:53:25.709

Moderator: on your mind

271

00:53:28.100 --> 00:53:34.149

Moderator: be any needs that aren't being met currently or something you think the school is doing really well either way

272

00:53:40.870 --> 00:53:45.149

A2.2: I I would commend the school for,

273

00:53:45.790 --> 00:53:51.859

A2.2: or I would commend, like the leaders in the [school] that I see that I think

274

00:53:51.980 --> 00:54:10.500

A2.2: do care about Phd Student Well being and are trying to start this change, I I know that I've made a lot of confidence, and I I don't want you to take them all as like. Oh, what a mess we're in um. I I don't want to come off that way. I know that there are people, and I think about like [dean]

275

00:54:13.860 --> 00:54:26.229

A2.2: like the people that work with him, and how much he tries to prioritize student well being, and it's not like everything that is happening is successful. But I feel

276

00:54:27.000 --> 00:54:30.229

A2.2: i'm. I'm grateful that we have

277

00:54:30.370 --> 00:54:38.619

A2.2: even one leader who's trying to make that a priority. And who's being an advocate for that. Um!

278

00:54:38.670 --> 00:54:52.949

A2.2: And so that is something i'm very thankful for that is present in the school. Um, I definitely have friends in other Phd programs where that does not even exist. And so

279

00:54:53.910 --> 00:55:01.730

A2.2: I don't want to say like there's progress to be made. But the progress that we have made is valuable too

280

00:55:13.110 --> 00:55:27.909

Moderator: thank you both for sharing all those recommendations. I think they're very, very useful, and um very thought provoking, so I really appreciate it. Um, do either of you have any other thoughts or suggestions that you would like to share. That, you think will be important for this study.

281

00:55:41.090 --> 00:55:57.469

A2.2: Um, I just want to make one last comment, and it's something it relates is just something A2.1 brought up earlier. Um, I think we've talked a lot about like the culture of the environment in our in our research labs and in in the divisions here, and that's

282

00:55:57.690 --> 00:55:59.899

A2.2: one area that

283

00:56:00.920 --> 00:56:15.770

A2.2: can either promote or take away from well being um. But A2.1 brought up um earlier about like financial stress being an issue for graduate students, and I, we haven't really talked about that, and I don't. I don't want to like

284

00:56:15.780 --> 00:56:25.500

A2.2: go down a tangent, but I think that is a very real stress for Phd students. It's something that I've heard from a lot of peers over the past.

285

00:56:25.840 --> 00:56:27.229

A2.2: Probably like

286

00:56:28.230 --> 00:56:33.340

A2.2: six months, maybe not, and maybe longer, but like

287

00:56:33.520 --> 00:56:37.060

A2.2: how financial challenges and dealing with,

288

00:56:37.120 --> 00:56:45.609

A2.2: You know a stipend that doesn't change very much or changes very, very slowly. Isn't. Always reflective of

289

00:56:45.990 --> 00:56:54.960

A2.2: the economic environment of [blinded city and state] is a is a very real source of stress for students.

290

00:56:54.980 --> 00:56:56.709

A2.2: Um, and

291

00:56:58.560 --> 00:57:13.940

A2.2: there's obviously many barriers that come with addressing that as an issue. But I I want to emphasize what a large issue it is for people, and it's definitely something that the school can do more to

292

00:57:13.960 --> 00:57:17.069

A2.2: come up with sustainable solutions for

293

00:57:17.200 --> 00:57:18.609

A2.2: um and that.

294

00:57:19.130 --> 00:57:20.100

A2.2: And

295

00:57:20.280 --> 00:57:22.459

A2.2: yeah, I'm just gonna stop there.

296

00:57:22.710 --> 00:57:40.929

A2.1: No, I I think that's super super important to mention. I'm so glad that you highlighted that more? Um, because it's like insane over the past year. How much things have changed! So I think when a lot of us started in the program. Our stipend wasn't great, but it was at least

297

00:57:40.940 --> 00:57:44.510

A2.1: near enough to like living wage for this

298

00:57:44.630 --> 00:57:55.420

A2.1: city, this part of the country that you it was going to be okay, you know you weren't gonna like have a great lifestyle. But it was manageable, and that has changed dramatically.

299

00:57:55.430 --> 00:58:07.939

A2.1: Um. And in addition to that, even though we've seen like I'm gonna put in quotes like a small raise. That was, I guess, communicated to us as addressing inflation.

300

00:58:07.950 --> 00:58:15.489

A2.1: That small change is so far below the amount that would be necessary to provide the same,

301

00:58:15.730 --> 00:58:22.410

A2.1: albeit low quality of life that was provided a couple of years ago in this program,

302

00:58:22.450 --> 00:58:41.389

A2.1: and this is something that is causing incredible financial stress to any student who does not have a partner who is making more than them. And I knew that it's affected people to the point of like increasing their um. I guess willingness to drop the program with a masters

303

00:58:41.400 --> 00:58:59.030

A2.1: or nothing. Um, So I think it's gonna directly impact the amount of like talent we're able to keep for lack of a better term in our program, and I think that the smartest graduate students like are very aware of, like what they're missing out on

304

00:58:59.040 --> 00:59:18.619

A2.1: because of the programs and ability to address the change in the cost of living conditions that has happened so quickly in this area, and I know that, of course, that affects all aspects of the school, and I know the school right now is probably not going super great, because they're also dealing with higher costs.

305

00:59:18.630 --> 00:59:36.009

A2.1: But I think, like a real concerted effort to find places where unnecessary money can be cut in order to support the workforce of the graduate side of our program is going to be completely necessary

306

00:59:36.020 --> 00:59:47.299

A2.1: to prevent a cycle of students leaving and acquiring. I I don't want to say, like I don't know like, of course, there's a lot of different things that go into the success of a graduate student.

307

00:59:47.500 --> 00:59:52.779

A2.1: But I think that a negative cycle will begin

308

00:59:52.910 --> 00:59:55.629

A2.1: in terms of retention, if that

309

00:59:55.640 --> 01:00:14.340

A2.1: the problem is not addressed, to say nothing of like how incredibly stressful it is for graduate students. But i'm actually very. I'm not only stressed for me. I'm stressed for the school because of it. Um! Just because I know it's such a big issue for students right now. So just yes, thank you for bringing that up. I think that is

310

01:00:14.350 --> 01:00:21.339

A2.1: super important to your trust when talking about well being um and people's ability to continue this program.

311

01:00:23.250 --> 01:00:24.609

A2.2: Yeah, I think it

312

01:00:24.940 --> 01:00:35.830

A2.2: it will really affect, like the most vulnerable members of our community, and I really relate to what A2.1 says said, where it's like.

313

01:00:36.010 --> 01:00:46.120

A2.2: I feel some stress for myself, but I feel a lot of stress for my peers, who, I know, will struggle more than I will, and

314

01:00:46.830 --> 01:01:01.549

A2.2: I think that the the school has a really important opportunity to step up and demonstrate support for those people that are most vulnerable. And I think that's something that the school has

315

01:01:02.340 --> 01:01:11.349

A2.2: at least said that they want to do, and they have said that that is a as like a goal for the strategic plan of

316

01:01:11.390 --> 01:01:22.720

A2.2: the School. And so this is really an opportunity for them to act on that as a stated goal, and to turn that into an action

317

01:01:26.410 --> 01:01:39.870

A2.1: and not just do the minimum, and then say that they've done something to help when it's the minimum, just to clarify to you, because i'm really afraid of that happening and continuing to happen where it's like. Oh, we gave you a raise That's like

318

01:01:39.880 --> 01:01:49.629

A2.1: eight points lower than inflation this year. So you're still using a ton of money compared to last year. It's just slightly less than you would have lost if we did nothing.

319

01:01:49.690 --> 01:01:53.450

A2.1: So just yeah, yes, one hundred percent, yes.

320

01:02:00.380 --> 01:02:30.079

Moderator: Thank you guys so much for sharing all of that. Um, just to be respectful of your time, we're gonna wrap up um. So thank you again for participating in this research. Your input. Is very valuable to our community, and i'm sure the findings of this study will help to inform and support future. Well-being efforts um if you happen to think of anything else you'd like to share. Um. There is a link in the outlook. Invite that you were sent. That would be to an anonymous survey. Um! And you'll be able to, and honestly,

321

01:02:30.090 --> 01:02:49.310

Moderator: anonymously provide any additional feedback. Um should you choose to. So if there's something you wish, you would have said in the session, but didn't get a chance to have forgot. Um, Please go ahead and use that link. Um! We will definitely be checking in on it. Um! But again thank you both so much for being here and sending your time with us. Um! We really really appreciate it.

322

01:02:51.110 --> 01:02:52.410

A2.1: Thank you.

323

01:02:52.850 --> 01:02:56.580

A2.2: Thank you. Have a good night.
