# Supplementary material for: Assessing factors that influence graduate student burnout in health professions education and identifying recommendations to support their well-being
Source: PLoS One. 2025 Apr 15;20(4):e0319857. doi: 10.1371/journal.pone.0319857 (PMC11999156; doi:10.1371/journal.pone.0319857)
Supplement: S4 File — (DOCX) [file pone.0319857.s004.docx]

**S4 File. ABD Focus Group Transcript**

10/6/2022

1

00:04:52.220 --> 00:05:09.929

Moderator: Hi! A1.1!

Moderator/A1.1: Hello! How's it going? How are you? Good? Okay, How are you?

Moderator: Great? Um, I think we're just waiting on um, One more participant, and then we'll um. Go ahead and get started like maybe one or two more minutes.

2

00:06:28.470 --> 00:06:39.779

Moderator: Okay, I want to respect your time. So i'm going to go ahead and like, do the introduction and um hopefully get started, and then um, the participant will hopefully join us as we talk. Is that all right with you.

3

00:06:39.820 --> 00:06:41.420

Yeah, Sounds good to me.

4

00:06:41.510 --> 00:07:04.249

Moderator: Okay, great. Um. So thank you for joining me today to discuss the Phd student. Well, being at pharmacy programs Uh, we greatly appreciate your time, and helping us to improve our school through the study. My name is Moderator and I'm pharmD student who will be conducting the focus group. Today. I work alongside [blinded] and [blinded] as well as [blinded]. Who oversees this project as our P. I.

5

00:07:04.260 --> 00:07:34.149

Moderator: Um. So this research project is entitled identifying structures that impact well, being Um and our irb number is twenty, one, one, six, two nine, and we know that you received a copy of the informed consent when signing up. But we just will briefly recap it. Now. Uh the focus group interview will be recorded, and all data will be de-identified prior to analysis and um dissemination, discussion and comments shared and the focus group will be identifiable by other focus. Group participants

6

00:07:34.160 --> 00:07:48.420

Moderator: and participants are reminded to not disclose any specific comments or dialogue with others outside the focus group meeting for the purpose of our discussion today we will be focusing on three domains, well being and burnout.

7

00:07:48.430 --> 00:08:04.989

Moderator: Um, and these domains will be or we're evaluated in the March twenty twenty-one well being baseline assessment, and which many have you participated in. Uh while the baseline assessment quantified the well being assessment, the purpose of the study is to further explore the qualitative factors that contribute to these domains.

8

00:08:05.000 --> 00:08:18.750

Moderator: Uh: so i'm just gonna briefly talk about uh these domains and how they're defined. So we can all be on the same page. Uh. So burnout is characterized by prolonged or repeated periods of stress, where a person begins to feel mentally exhausted by their tasks,

9

00:08:18.760 --> 00:08:31.400

Moderator: and well Being  describes uh

10

00:08:31.510 --> 00:08:36.640

Moderator: and well being is characterized as a state of being happy, healthy, and prosperous.

11

00:08:36.760 --> 00:08:41.740

Moderator: Uh, so do you have any questions before we get started with Um. My questions.

12

00:08:42.679 --> 00:08:58.629

A1.1: Yeah, I guess I was wondering what you were hoping to do with the data that you collect. Are you planning to like implement changes within UNC

13

00:08:58.640 --> 00:09:13.730

Moderator: Yes we will be, like I said all information will be deidentified, and um I'm using this for my RASP project within our curriculum. Um. But the information is also going to go to the well-being committee, which will then um use the information to hopefully provide um recommendations to our school.

14

00:09:13.780 --> 00:09:15.230

A1.1: Okay, Very cool.

15

00:09:15.320 --> 00:09:16.260

Moderator: Yeah.

16

00:09:16.930 --> 00:09:35.910

Moderator: So this is actually the um fourth group of um participants that we're doing a part of this study. So I think last year it got started with faculty members and pharmD. Students. And then this year we're completing Phd. Students and um staff members in the school.

17

00:09:36.040 --> 00:09:44.720

A1.1: Nice. I think it's really good that you guys are doing this

Moderator: Um, thank you. Yes. I'm excited to learn about your experience and well-being at our school.

18

00:09:45.470 --> 00:09:53.150

Moderator: Uh. So the first question I have is what factors positively affect your well-being and bring and or bring your you fulfillment.

19

00:09:53.770 --> 00:10:01.939

A1.1: Yeah, um. So should this be tied specifically to the the school, I guess,

Moderator: or just you in general, really … your experiences.

20

00:10:02.000 --> 00:10:04.689

A1.1: okay,

21

00:10:04.780 --> 00:10:12.270

A1.1: Yeah. So I would say, just in general, Um, having people to relate to and people to communicate with. So

22

00:10:12.340 --> 00:10:30.680

A1.1: having, like a a supportive environment, be like the PI, your lab mates at work, and then also at home. So I like having friends spouse significant other a pet like people you can connect to, and like vent with a little bit very helpful.

23

00:10:30.940 --> 00:10:32.170

A1.1: Um,

24

00:10:35.640 --> 00:10:42.890

A1.1: I would say, having like workwise having an understanding PI um someone who is like

25

00:10:43.150 --> 00:10:47.789

A1.1: attentive to your needs and like willing to make changes um

26

00:10:48.570 --> 00:10:51.670

A1.1: to sort of adapt to your needs of the students.

27

00:10:52.060 --> 00:10:55.370

A1.1: Um! somebody listens to you. Um!

28

00:10:55.890 --> 00:10:58.489

A1.1: And take your ideas into consideration,

29

00:10:58.940 --> 00:11:05.280

A1.1: which I don't think is always the case uh for someone you know in in like a superior role.

30

00:11:06.050 --> 00:11:07.290

A1.1: Um:

31

00:11:10.910 --> 00:11:15.619

A1.1: yeah, as a Phd student like lab work can take

32

00:11:16.150 --> 00:11:23.270

A1.1: a lot out of you, uh like, physically and mentally like sometimes you're here for a very long period of time. Um,

33

00:11:23.320 --> 00:11:25.390

A1.1: or your you like you just

34

00:11:25.550 --> 00:11:32.350

A1.1: you don't always work a forty hour week um almost. In fact, you like almost never do um so

35

00:11:32.430 --> 00:11:33.560

A1.1: um

36

00:11:34.660 --> 00:11:39.580

A1.1: having having like people around you who at least recognize that, and like you're able to

37

00:11:40.010 --> 00:11:42.850

A1.1: commiserate with or hope, like you know,

38

00:11:43.830 --> 00:11:48.309

A1.1: share the load if if you will. Um, hopefully, that helps.

39

00:11:48.820 --> 00:11:49.850

Moderator: Yeah,

40

00:11:52.840 --> 00:12:10.970

A1.1: I think those are the things like just having like a a network of people who understand you.

Moderator: Yeah. So I hear a lot about that. You're saying relationships are really important and helping maintain your well being. Um, How do you think um

41

00:12:11.020 --> 00:12:12.649

Moderator: what helps you to make

42

00:12:12.720 --> 00:12:15.659

Moderator: you feel valued um

43

00:12:16.350 --> 00:12:20.020

Moderator: at your job, or at school, or in general?

44

00:12:22.680 --> 00:12:25.060

A1.1: Um, let's see.

45

00:12:26.190 --> 00:12:43.250

A1.1: Well, this may sound a little cheesy, but like words of affirmation from a PI, or a lab mate to go a really long way. Um! So like having a bit of reassurance that, like you're doing. You're doing fine, I know. When I was very like early on in my dissertation I was.

46

00:12:43.270 --> 00:12:51.669

A1.1: I have tons of imposter syndrome, and I still do I'm just like better at managing it now. But, like you know, knowing that you're not the only one that feels that way

47

00:12:51.760 --> 00:12:54.340

A1.1: uh really helps, and

48

00:12:54.530 --> 00:13:04.199

A1.1: in terms of like helping you feel valued like knowing that, like you know, you're doing fine like you've done well here that can go a very long way. And then also like,

49

00:13:05.840 --> 00:13:06.810

A1.1: well,

50

00:13:06.880 --> 00:13:10.719

A1.1: you're saying like feeling valued specifically. Um.

51

00:13:10.990 --> 00:13:14.160

Moderator: Or does that help to improve your well being

52

00:13:15.020 --> 00:13:17.990

A1.1: definitely Yeah.

53

00:13:18.200 --> 00:13:19.450

A1.1: Um,

54

00:13:21.910 --> 00:13:26.239

A1.1: yeah. And honestly that sense of accomplishment you get when you

55

00:13:26.400 --> 00:13:30.470

A1.1: you finish something related to your research or your dissertation. So like

56

00:13:31.050 --> 00:13:34.689

A1.1: finishing up a class or an exam um

57

00:13:34.960 --> 00:13:41.999

A1.1: like when I submitted my first paper that felt like a huge milestone to me.

58

00:13:42.400 --> 00:13:47.810

A1.1: So just like getting through my preliminary exams, like meeting those milestones Also, it's really helpful.

59

00:13:47.940 --> 00:13:49.220

A1.1: Yeah.

60

00:13:52.040 --> 00:13:53.030

A1.1: Yeah.

61

00:13:53.150 --> 00:13:57.100

Moderator: And do you have like anything

62

00:13:57.440 --> 00:14:03.419

A1.1: like exercise or hobbies? Um: That may help to improve your well-being.

63

00:14:03.560 --> 00:14:05.960

A1.1: Yeah, absolutely. Um.

64

00:14:06.040 --> 00:14:11.730

A1.1: So I really like spending time outside. I like to backpack um spend time in nature, so

65

00:14:11.900 --> 00:14:14.009

A1.1: I go on backpacking trips

66

00:14:14.530 --> 00:14:20.819

A1.1: once every month or so for a weekend, and, like I love doing that. Um,

67

00:14:21.070 --> 00:14:22.689

A1.1: I really enjoy

68

00:14:22.710 --> 00:14:28.069

A1.1: uh cooking so like in your recipes trying to stuff out um.

69

00:14:29.220 --> 00:14:36.820

A1.1: That's always something I go to. It's like Really, it's big in my family, like if you're from the South, you know. Um,

70

00:14:37.170 --> 00:14:44.729

A1.1: uh, I also really like to draw and paint um. So I do that sometimes my free time

71

00:14:44.850 --> 00:14:46.500

A1.1: and I like to read

72

00:14:46.580 --> 00:14:53.679

A1.1: so that sometimes I like, listen to audiobooks and doing bench work and stuff. Um: Yeah. So yeah,

73

00:14:54.340 --> 00:14:57.340

A1.1: awesome. Yeah, And that makes a positive impact for sure.

74

00:14:57.470 --> 00:14:58.630

Moderator: Mhm

75

00:15:00.210 --> 00:15:13.069

Moderator: Okay? Well, now, we're going to move on to uh the the question that you may have more thoughts about um. But what factors negatively affect your well being and or cause burnout.

76

00:15:15.210 --> 00:15:16.400

A1.1: Yeah,

77

00:15:16.450 --> 00:15:17.560

A1.1: um.

78

00:15:19.310 --> 00:15:23.909

A1.1: I think a a big one for me, especially during the pandemic was like

79

00:15:24.800 --> 00:15:29.310

A1.1: lack of transparency and confusion on whether or not you're

80

00:15:29.460 --> 00:15:33.129

A1.1: on the right path. Um, or if you were like

81

00:15:33.980 --> 00:15:43.820

A1.1: meeting the the markers that you needed to meet as a student like. They're just like no one had any level of certainty at all during the pandemic, right? So like

82

00:15:43.900 --> 00:15:53.069

A1.1: that's normal, I guess, at least for that time. But it was. It had a really big negative impact on my well being as a student um feeling like You're

83

00:15:53.800 --> 00:16:02.050

A1.1: just like in free Fall, and you can't like you can't do your research like you feel crummy. So you can't like writing is really difficult. And

84

00:16:02.410 --> 00:16:03.660

A1.1: um

85

00:16:04.710 --> 00:16:08.269

A1.1: yeah. So like, I guess, like a lack of clear

86

00:16:08.380 --> 00:16:15.249

A1.1: your like goals or um your expectations. And in the time where like things are certain.

87

00:16:15.270 --> 00:16:28.349

Moderator: Yeah, do you feel like that was caused by just the pandemic and like what you said like just the whole fact that that…

88

00:16:28.610 --> 00:16:35.510

A1.1: it's also just a trademark of kind of grad school in general, like everybody, has such a different

89

00:16:35.640 --> 00:16:39.800

A1.1: like individual path in their research. Um, So they're like

90

00:16:39.820 --> 00:16:50.299

A1.1: there's no like rubric you can follow for a Phd. Right like I mean, they're kind of like your classes and your exams, but like beyond that, it's like you'll figure it out. Um,

91

00:16:50.350 --> 00:16:54.530

A1.1: and that's just how it is. It kind of goes with the Territory. But, uh,

92

00:16:54.860 --> 00:17:01.410

A1.1: just it. It really does do a number on you in terms of like stress. Um! And I can't think of a like.

93

00:17:01.510 --> 00:17:07.810

A1.1: I think that's probably the way it it always has been um part of why it's so stressful.

94

00:17:08.109 --> 00:17:09.319

A1.1: Um,

95

00:17:10.790 --> 00:17:11.859

A1.1: yeah,

96

00:17:14.430 --> 00:17:18.230

A1.1: I would say like something that recently has been

97

00:17:18.720 --> 00:17:27.500

A1.1: more of an issue. And I think you know, students are doing a better job like we're trying to bring that to people's attention. More is um

98

00:17:27.970 --> 00:17:35.330

A1.1: compensation for grad students. So I know that's been a big topic recently. But um. The

99

00:17:35.520 --> 00:17:39.729

A1.1: The compensation that you know students receive is not

100

00:17:40.110 --> 00:17:50.820

A1.1: increased at  the level that goes with the rate of inflation, and the cost of living here in [city] has increased so much. Um, I know there are like students in our program who are paycheck the paycheck.

101

00:17:51.060 --> 00:17:52.170

A1.1: Um.

102

00:17:52.950 --> 00:17:58.520

A1.1: So I I think that has an impact for sure. Yeah, I know when I was in my first year like I was

103

00:17:58.880 --> 00:18:07.990

A1.1: um. I was like supporting my then fiancé at the time, and I was on like a first year grad student stipend, and I was like living paycheck to paycheck, and that’s stressful.

104

00:18:08.040 --> 00:18:09.130

Moderator: Um,

105

00:18:09.930 --> 00:18:10.820

A1.1: So

106

00:18:11.410 --> 00:18:14.160

A1.1: that can have an impact for sure.

107

00:18:14.390 --> 00:18:15.550

A1.1: Um,

108

00:18:19.470 --> 00:18:22.060

Moderator: I know you mentioned um

109

00:18:22.370 --> 00:18:26.050

earlier talking about. You know you

110

00:18:26.410 --> 00:18:36.680

Moderator: work more than forty hours a week. Does that impact your well-being?

Yeah, yeah, Absolutely that's like like I said, that's like like when you

111

00:18:37.030 --> 00:18:43.120

A1.1: enter into grad school. It's kind of the expectation, you know, when maybe it shouldn't have to be

112

00:18:43.160 --> 00:18:44.630

A1.1: uh,

113

00:18:44.670 --> 00:18:55.240

A1.1: but you know, like realistically, if you want to get through the program and succeed like within a timely manner, you you just have to. There's kind of no other way around that which

114

00:18:55.320 --> 00:19:00.909

A1.1: I don't know. If there's ways we can change that to make it better. I'm sure we like. There probably are, I mean.

115

00:19:01.060 --> 00:19:10.130

A1.1: But I think we're just so used to thinking that that's the the norm and the expectation that people probably don't think very hard about how to change it. But

116

00:19:10.220 --> 00:19:11.370

Moderator: yeah,

117

00:19:12.310 --> 00:19:15.990

A1.1: it does. Uh take a toll for sure.

118

00:19:16.090 --> 00:19:34.409

A1.1: Um, especially like early on when you're in courses. Um, you're trying to do course work and lab work at the same time, and you're constantly worried about like if I don't do enough. You know, if i'm not doing enough course work or lab work, you know, spending time on one it's taking away from time on the other.

119

00:19:34.530 --> 00:19:35.900

A1.1: Uh: So

120

00:19:36.310 --> 00:19:43.149

A1.1: I would say earlier, like before I was like ABD, that was a a source of stress.

121

00:19:43.230 --> 00:19:44.510

Yeah, Yeah,

122

00:19:44.780 --> 00:19:47.950

Moderator: yeah, Can you talk a little bit more about that, like the

123

00:19:48.030 --> 00:19:53.220

Moderator: um, maybe specific curriculum or research stressors?

124

00:19:55.330 --> 00:20:07.750

A1.1: Yeah. So I think the the [Division] program, anyway, like we have a pretty rigorous curriculum. It's quite a few hours that you're expected to do um in your first two years,

125

00:20:08.150 --> 00:20:13.319

A1.1: and then to do that like on top of your research, and to feel like you're able to make

126

00:20:13.560 --> 00:20:17.840

A1.1: significant strides is really difficult. Um,

127

00:20:18.850 --> 00:20:35.060

A1.1: and you know, like I think every I think every division has some version of like a seminar that every student has to do every year. So like I know. In my first year. I was absolutely terrified of that. It was like, I don't know what i'm doing. I don't know, like how i'm going to get through

128

00:20:35.320 --> 00:20:39.680

A1.1: uh getting this project, and, like be able to present it in a way that's,

129

00:20:39.920 --> 00:20:46.549

A1.1: you know, knowledgeable enough to be in front of all my peers and all my faculty asking me all these questions. So

130

00:20:46.950 --> 00:20:50.819

A1.1: it's just the the first year Imposter syndrome and feeling like you're not

131

00:20:51.390 --> 00:20:55.680

A1.1: uh You're not up to par with everyone else, which is,

132

00:20:55.840 --> 00:20:57.860

A1.1: you know, like now that i'm

133

00:20:57.920 --> 00:21:08.009

A1.1: further along, I know that, like you can't directly compare yourself to people who are further along the program that you know It's not a good. It's not good. Um: Um:

134

00:21:09.390 --> 00:21:10.570

A1.1: yeah,

135

00:21:11.970 --> 00:21:15.250

Moderator: yeah. Do you think that your wellness

136

00:21:15.340 --> 00:21:22.140

Moderator: um improved and like your resiliency improved after becoming ABD status.

137

00:21:22.290 --> 00:21:28.539

A1.1: Absolutely. Absolutely. Yeah, I feel like I had a path. I had a plan.

138

00:21:28.710 --> 00:21:35.439

A1.1: I've got all I've got my specific aims written out. My Dissertation Committee approved them. I just need to do it. Um,

139

00:21:36.070 --> 00:21:37.910

A1.1: yeah, I would after my.

140

00:21:39.110 --> 00:21:44.600

A1.1: I guess it was like out at the end of my third year. So my fourth year that I did my um

141

00:21:44.860 --> 00:21:57.049

A1.1: pre lims. After that I felt like worlds better. I was so stressed going into that um, and like It's not that I don't have stress now, but it like, I feel like I have more clarity.

142

00:21:57.260 --> 00:21:58.420

A1.1: Um,

143

00:21:59.320 --> 00:22:03.889

Moderator: yeah, yeah. And do you feel like the uh

144

00:22:04.320 --> 00:22:15.230

Moderator: the stress around like the pre lims and stuff had to do, and it's correlated with like the impostor syndrome that you felt, or just without the like clear path.

145

00:22:15.480 --> 00:22:18.340

A1.1: I think it's a little bit of both like um.

146

00:22:18.380 --> 00:22:20.559

A1.1: The imposter syndrome is like

147

00:22:21.060 --> 00:22:27.229

A1.1: a huge part of it, because you have like you've come up with all these aims on your own, and you're thinking like

148

00:22:28.160 --> 00:22:31.189

A1.1: I don't know what i'm doing. I don't know if this is right like.

149

00:22:31.350 --> 00:22:35.149

A1.1: Are they just going to tear it to pieces? Um.

150

00:22:36.530 --> 00:22:44.879

A1.1: So that yeah, the imposter syndrome is a huge part of it. Um, feeling like maybe you're not like you're going to this, and still don't know what you're doing.

151

00:22:45.060 --> 00:22:46.310

A1.1: Um!

152

00:22:47.480 --> 00:22:49.250

A1.1: What was the other part you said

153

00:22:49.820 --> 00:23:04.780

Moderator: like? Uh, I hear you talk a lot about um like feeling like you don't have a clear path, and then, like once you got to Abd, it It was like, Okay, I have my clear path. I know what i'm doing. Everything is kind of set.

154

00:23:04.940 --> 00:23:16.489

A1.1: Yeah. So okay, yeah, I think those two things kind of go hand in hand. It's like you're trying to come up with the path on your own by designing your your aims,

155

00:23:16.510 --> 00:23:25.449

A1.1: and not knowing if it's like you're doing the right thing or not, or if, like what you're doing is feasible, or um worthwhile to do

156

00:23:25.870 --> 00:23:27.050

A1.1: um,

157

00:23:27.190 --> 00:23:31.690

A1.1: and the uncertainty about like whether or not you can actually like um

158

00:23:32.280 --> 00:23:33.380

A1.1: sort of

159

00:23:34.860 --> 00:23:45.650

A1.1: finish, like everything you intend to do within a reasonable amount of time. Usually your your committee helps you with that sort of thing. If you're like doing like pie in the sky kind of aims. But uh,

160

00:23:46.790 --> 00:23:55.050

A1.1: yeah, I think like when you start out like you have these really big ideas of like what you can do, what you're gonna do. And then like that, you get through grad school like

161

00:23:55.560 --> 00:24:02.280

A1.1: you bit like your expectations become a little bit more realistic in terms of like, Okay, here's what I can feasibly do.

162

00:24:02.590 --> 00:24:03.720

A1.1: Um,

163

00:24:03.970 --> 00:24:08.809

A1.1: with a most amount of time. So it's just like a big adjustment, I guess.

164

00:24:08.900 --> 00:24:12.409

Moderator: Yeah, is the time to uh

165

00:24:13.420 --> 00:24:26.559

Moderator: completion of your Phd. Also, like impact your well being like trying to maintain a certain timeline that um for your own personal goals.

166

00:24:26.580 --> 00:24:33.880

A1.1: It certainly can be um like, especially like when you're leading up to your preliminary exam, like the

167

00:24:34.040 --> 00:24:36.130

A1.1: sort of model is like

168

00:24:36.400 --> 00:24:51.460

A1.1: um. So you finish up your classes, you' to do your prelim defense like end of your two, or during your three, and then you graduate by the end of year five, and if you don't do that, then something's wrong like, or you've messed up somehow. Um,

169

00:24:51.950 --> 00:24:58.100

A1.1: I think I have that mentality starting out like for sure, and I still like fight it a little bit.

170

00:24:58.200 --> 00:25:03.730

A1.1: Um, i'm not um. I feel a little bit more secure in my place in the

171

00:25:03.900 --> 00:25:16.839

A1.1: thesis in the dissertation trajectory now than I used to. But it's taken me a long time to get comfortable with that, because I felt like I was really behind leading into my preliminary exam

172

00:25:16.860 --> 00:25:20.580

A1.1: my third year was basically the pandemic, and that was just like

173

00:25:20.660 --> 00:25:29.279

A1.1: it was a Really, it was not a good year. Um. So like my mental health, suffered, and, like my time to getting my pre lim done,

174

00:25:29.370 --> 00:25:35.189

A1.1: was affected as a result. Um! And like it, was hard not to feel like that failure when, uh

175

00:25:35.390 --> 00:25:38.919

A1.1: you feel like you're falling behind.

176

00:25:39.820 --> 00:25:53.899

Moderator: Did you feel like you were falling behind in regard to um, the traditional timeline, or in regard to your peers, and where you um were in your project compared to your peers.

177

00:25:54.800 --> 00:26:05.379

A1.1: Yeah, I guess it probably depends on the person, but I think for me. It was like, Oh, i'm not meeting expectations. I'm not meeting my PI's expectations, and that I’m not meeting the department's expectations.

178

00:26:05.770 --> 00:26:19.799

A1.1: They're all gonna think less of me. They're gonna kick me out, that kind of thing and like. And then the the peers, you know, being finished or like finishing up was like to me was like, fuel to the fire. It's like i'll look. They're finishing. Why can't, What like?

179

00:26:19.880 --> 00:26:25.689

A1.1: Why haven't you finished yet? You know. So yeah, Yeah, that makes sense.

180

00:26:25.950 --> 00:26:27.090

Moderator: Um.

181

00:26:27.320 --> 00:26:34.450

Moderator: And I know you mentioned, you know, having a good relationship with your pi um and mentors

182

00:26:34.580 --> 00:26:44.229

Moderator: helps your well-being is there any thing that could contribute, and that realm to uh negatively affect your well being.

183

00:26:45.790 --> 00:27:01.279

A1.1: Yeah, I would say, like having like, not like poor communication with your PI generally like if i'm not able to like, reach my PI in a timely manner, and i'm feeling lost like, especially early on that was a big source of stress for me.

184

00:27:01.430 --> 00:27:02.670

A1.1: Um,

185

00:27:03.590 --> 00:27:06.120

A1.1: you know, like you get better at managing that,

186

00:27:06.370 --> 00:27:18.219

A1.1: and you get more experience as you move along in the program. And also you just generally like, have less questions because you learn stuff, um, or you're able to figure it out on your on your own more

187

00:27:18.450 --> 00:27:23.959

A1.1: um, so like lack of communication, lack of clarity from the PI, or like

188

00:27:24.890 --> 00:27:25.920

A1.1: just

189

00:27:26.770 --> 00:27:32.359

A1.1: it can be very discouraging. Early on. If you're learning, you're just learning something, and uh

190

00:27:32.710 --> 00:27:38.139

A1.1: the feedback you get from your peers or your pi is all negative, you know.

191

00:27:45.350 --> 00:27:48.379

A1.1: Yeah, and not to say that constructive critism is not a good thing, it’s a very good thing.

Moderator: But with the positives, right?

192

00:27:48.400 --> 00:27:49.280

A1.1: Yeah.

193

00:27:49.330 --> 00:27:50.270

Moderator: Yeah.

194

00:27:51.320 --> 00:27:57.379

Moderator: So anything else that you can think of that negatively affects your well being or leads to burnout.

195

00:28:05.360 --> 00:28:10.739

Moderator: I guess you mentioned.

A1.1: Oh, I was gonna say, I think a big thing that led to like

196

00:28:12.040 --> 00:28:21.609

A1.1: stress for me was feeling like I was alone on that uh feeling like I was the only one falling behind. I was.

197

00:28:22.520 --> 00:28:30.060

A1.1: Everyone else had it together, and I didn't. So basically imposter syndrome. Um: Yeah. So yeah,

198

00:28:30.150 --> 00:28:48.579

A1.1: that like feeling yeah, just feeling like you're alone. And you're not. You're not up to par for some reason. Um, because for me was the biggest source of like mental stress. Um, yeah.

Moderator: And you mentioned your committee members that they could maybe help you um

199

00:28:49.740 --> 00:28:50.700

Moderator: like,

200

00:28:51.220 --> 00:29:07.189

Moderator: solidify your aims. How does um? How do you feel that committee members help or hurt your well being or that structure?

A1.1: Yeah, Well, I think it probably depends on the individuals you have on your committee. Um!

201

00:29:07.200 --> 00:29:15.009

A1.1: I I really like my committees. They're very like they're very supportive. Um! And their feedback is always constructive. Like they're not.

202

00:29:15.330 --> 00:29:20.880

A1.1: They're always very like polite and constructive with their feedback for me, even if they have like something

203

00:29:21.740 --> 00:29:26.240

A1.1: like things to improve. Um, let's say um.

204

00:29:26.960 --> 00:29:41.839

A1.1: So for me it's been very helpful like they help me like rein and stuff that's like. Whoa! This is gonna take way too much time to do um, or Oh, have you thought about this like for this aim? I think this would be really good to pursue. Um,

205

00:29:42.400 --> 00:29:51.799

A1.1: and I like to think about how a dissertation committee is supposed to function like they're supposed to be a source of knowledge and expertise to help guide you. Um.

206

00:29:52.550 --> 00:30:01.880

A1.1: So for myself it's been. I think it's been pretty positive. But I I always get really nervous going in the meetings, of course. But Um: yeah, yeah,

207

00:30:02.210 --> 00:30:05.519

A1.1: Okay, Yeah. Good.

208

00:30:05.540 --> 00:30:13.480

Moderator: Um. So what recommendations would you suggest to improve graduate student Well being at the school?

209

00:30:15.840 --> 00:30:18.169

A1.1: great question? Um,

210

00:30:21.780 --> 00:30:24.160

A1.1: I think like encouraging

211

00:30:25.830 --> 00:30:32.660

A1.1: connection within the school, like helping students not feel alone, so like how encouraging

212

00:30:33.230 --> 00:30:35.929

A1.1: Phd students interact with each other

213

00:30:36.020 --> 00:30:51.599

A1.1: more and just to interact with the school more in general, I think. Um, You've probably heard this from others, but the Phd students, I think a lot of times feel kind of siloed in what they're doing, and like I feel like disconnected from pharmacy students and um faculty that teach within the pharmacy school in terms of pharmacy students and phd program. Feels very separate

214

00:31:01.550 --> 00:31:04.109

A1.1: um, and I know they've tried to make

215

00:31:04.360 --> 00:31:09.380

A1.1: changes to kind of improve that. But I I I don't think it's stuck yet.

216

00:31:10.040 --> 00:31:11.170

A1.1: Um,

217

00:31:13.030 --> 00:31:22.049

A1.1: yeah, I I think encouraging like community among students would be the biggest thing. Yeah, yeah,

218

00:31:22.340 --> 00:31:25.609

Moderator: what do you think is um

219

00:31:27.010 --> 00:31:32.839

Moderator: successful with what the school is doing now. That helps um improve. Well being,

220

00:31:35.450 --> 00:31:38.550

A1.1: I think that's great that we have uh

221

00:31:38.670 --> 00:31:44.020

A1.1: sort of the well-being days um, that [the Dean] has implemented.

222

00:31:44.330 --> 00:31:57.329

A1.1: Um! I don't know how evenly that's actually enacted across the Ph. D program. But I know that my PI like respects that time, and that's great. I love It's nice to have that little bit of time to breathe um,

223

00:32:00.780 --> 00:32:02.630

A1.1: and I think uh,

224

00:32:02.950 --> 00:32:04.120

A1.1: you know, like

225

00:32:04.620 --> 00:32:11.310

A1.1: we have some good like affiliate and student organizations for Phd. Students, and I think help foster that community.

226

00:32:11.500 --> 00:32:21.489

A1.1: Um! So I think those are good things and like they're mostly student led. But I think like kind of encouraging involvement. And those kind of things can be really helpful. Uh sometimes.

227

00:32:22.440 --> 00:32:23.700

A1.1: And

228

00:32:24.840 --> 00:32:26.100

A1.1: yeah, I think

229

00:32:26.670 --> 00:32:27.880

A1.1: we haven't had.

230

00:32:27.920 --> 00:32:30.869

A1.1: We've had a few of these in the past, but I think, having like

231

00:32:31.450 --> 00:32:40.260

A1.1: kind of like encouraging people to like meet together socially, and a relaxed atmosphere, I think, is always nice. Um!

232

00:32:41.200 --> 00:32:59.669

A1.1: And it's hard to get people to want to do stuff like that all the time. So it's. It's easy for people to say that, but actually like it's really hard to like actually create a genuine community when it's like coming from the higher ups, you know. Um, I don't know It's really tricky. Um,

233

00:33:01.250 --> 00:33:10.330

A1.1: I wish they had a better answer for like what we could do for that. But I Don't:

Moderator: Yeah. Um: yeah, it's hard like you said.

234

00:33:10.520 --> 00:33:16.500

Moderator: I know earlier you mentioned um like compensation as being a source of uh

235

00:33:17.080 --> 00:33:26.439

Moderator: like negative factors that would affect your well being. Um! What recommendations would you provide around compensation?

236

00:33:26.600 --> 00:33:30.239

A1.1: Um, I would say, like for starters uh

237

00:33:30.830 --> 00:33:40.499

A1.1: Phd Students, and all the divisions need to be paid like equally with other Phd students entering the program from tips or not to uh [other program]

238

00:33:41.040 --> 00:33:41.990

A1.1: uh,

239

00:33:42.380 --> 00:33:46.589

A1.1: for the sake of equity like it, I don't think it's right that a PhD student with the same level of experience who is just coming from a different department is getting paid more than another

240

00:33:53.710 --> 00:33:54.830

A1.1: um!

241

00:33:56.100 --> 00:33:59.659

A1.1: And then I would say to like, look at the cost of living, and make sure that, like

242

00:33:59.830 --> 00:34:05.829

A1.1: the salary that you're offering is oh, like aligned with the current cost of living.

243

00:34:05.890 --> 00:34:10.270

A1.1: Uh, with where you are. And I don't, and I don't mean to complain, because I know, like

244

00:34:10.350 --> 00:34:18.069

A1.1: in our program like at Unc. Uh we're we're compensated very well. I like it based on like the national average,

245

00:34:18.210 --> 00:34:20.720

A1.1: and i'm aware of that. Um.

246

00:34:21.120 --> 00:34:32.099

A1.1: But I I I I still think you should like we should recognize that that average is very low and does not meet the cost of living for most students. So

247

00:34:32.710 --> 00:34:33.810

Moderator: yeah,

248

00:34:34.020 --> 00:34:36.969

Moderator: Are there any other needs that are being met?

249

00:34:51.560 --> 00:34:53.410

A1.1: I feel like, uh,

250

A1.1: where i'm at right now. In my dissertation I feel I feel pretty like supported um. But i'm I'm entering into my fifth year, and i'm like I know what I need to do.

251

00:35:03.060 --> 00:35:08.720

A1.1: Yeah, I think it's easy for me to say that. Um, from where i'm sitting um

252

00:35:10.440 --> 00:35:17.279

Moderator: anything that you think of from like earlier in your time, maybe even when you were pre-ABD

253

00:35:24.300 --> 00:35:25.689

A1.1: yeah, I

254

00:35:30.280 --> 00:35:33.359

A1.1: I think It's like having good

255

00:35:33.540 --> 00:35:38.979

A1.1: like solid communication with all the students about what expectations are. Um,

256

00:35:41.170 --> 00:35:44.349

A1.1: honestly, I felt like going into my uh

257

00:35:45.270 --> 00:35:51.820

A1.1: my like uh finishing course work my written exam and my oral exam.

258

00:35:52.090 --> 00:35:53.200

A1.1: Um.

259

00:35:53.840 --> 00:36:07.719

A1.1: There was a lot of like uncertainty around that, like I think that like expectations and like how that normally goes probably could have been communicatied a lot better. Um, and like I said, I figured it out. But, like I, you know, it's a lot of like,

260

00:36:08.350 --> 00:36:14.489

A1.1: stress. Yeah, a lot of stress around that. So that probably could have been avoided.

261

00:36:15.570 --> 00:36:16.810

A1.1: Uh-huh.

262

00:36:18.700 --> 00:36:19.720

A1.1: Yeah.

263

00:36:23.250 --> 00:36:33.669

A1.1: I think, Yeah. It's. It's just getting like PhD students to talk to each other and like like, so that they don't feel alone in their confusion or struggles is one of the biggest things you can do

264

00:36:38.990 --> 00:36:49.900

Moderator: yeah. Yeah. So is there anything um else, any other thoughts or suggestions that you think um would be important to share for uh this study.

265

00:37:01.040 --> 00:37:08.490

A1.1: Uh, I guess I would just say that I appreciate the efforts that the the school is trying to make to improve our wellbeing. Um!

266

00:37:10.250 --> 00:37:11.450

A1.1: And I

267

00:37:11.830 --> 00:37:13.459

A1.1: like I I

268

00:37:14.090 --> 00:37:16.350

A1.1: I don't want to come off as like.

269

00:37:18.410 --> 00:37:22.160

A1.1: ungrateful for anything. Um! But I I want to like

270

00:37:22.330 --> 00:37:30.939

A1.1: I guess it's important to understand, like the difference between like appearing to like, provide support and actually providing support. Is it like,

271

00:37:31.680 --> 00:37:39.100

A1.1: I think sometimes, and this is not always the case. Um, like this is a prime example of you guys making good strides and like

272

00:37:39.380 --> 00:37:44.119

A1.1: making an effort to like connect with people and figure out like how you can prove um.

273

00:37:45.120 --> 00:37:49.399

A1.1: Sometimes the I think, like things are done just to give

274

00:37:49.860 --> 00:37:55.970

A1.1: so like, Cross that off the list like, okay, we've offered this resource. Um, but maybe like

275

00:37:56.580 --> 00:38:04.260

A1.1: there's just not a lot of like talk around it, or like. There's still a a stigma there around, actually accessing the resource. Um!

276

00:38:04.700 --> 00:38:12.850

A1.1: So it's not enough to just like, say that you have the resource or say what you're doing, you need, to actually like, encourage people to use it and like, demonstrate that.

277

00:38:13.140 --> 00:38:15.439

A1.1: Yeah, this is, you know

278

00:38:15.590 --> 00:38:16.520

A1.1: the

279

00:38:16.940 --> 00:38:27.010

A1.1: I think maybe I hope you understand what i'm saying? Um, yeah, you haven't.

Moderator: Do you have an example of like something that you feel the school was like, Hey,

280

00:38:27.350 --> 00:38:36.140

Moderator: here's this like band aid for your gaping wound. You know, if you have an example that you're thinking of. Yeah, um,

281

00:38:36.310 --> 00:38:40.080

A1.1: i'm. I'm thinking of last year when there were a lot of like mental health issues in undergraduate students on campus. You know we had a lot of incidences with students self-harming and taking their own lives which is just awful and

282

00:38:54.140 --> 00:38:55.290

A1.1: um

283

00:38:56.230 --> 00:39:01.439

A1.1: I All I ever heard about it was like an email, you know, in the school, and

284

00:39:02.230 --> 00:39:11.010

A1.1: I think we need to take more time to recognize that that's a sign that things are not good. Um: yeah,

285

00:39:12.150 --> 00:39:30.919

A1.1: Same thing with [controversial university event] to the school for, and I think it was. It was very easy for us to not think about it until, like the very end, Like with [controversial university event] like we uh we kind of just didn't talk about it. Chose not to like discuss it until right at the very end, when it was like a national headlines.

286

00:39:30.930 --> 00:39:35.290

A1.1: And you're like Oh, we should say something. We should do something. Yeah. Yeah. So

287

00:39:36.080 --> 00:39:38.379

A1.1: yeah. And then, yeah,

288

00:39:39.920 --> 00:39:44.839

A1.1: I love that we offer like things for mindfulness and like mental health

289

00:39:45.090 --> 00:39:49.619

A1.1: and like have workshops for that. But I also want to recognize that, like mindfulness

290

00:39:50.680 --> 00:40:03.079

A1.1: shouldn't be the solution to like a system that is like causing stress.  You should change the system right like you Shouldn't. Just be like Here's tools to cope with the

291

00:40:03.240 --> 00:40:10.109

A1.1: really stressful system that we have instead of like the stressors, you know. So

292

00:40:10.220 --> 00:40:13.120

A1.1: thinking about the root cause and yeah,

293

00:40:13.430 --> 00:40:30.819

Moderator: yeah, Oh, yeah. So there's anything that um the school has done when you're like. Oh, wow! Like I feel. I'm really glad that they addressed this or um. They did address the root cause for this situation,

294

00:40:32.450 --> 00:40:36.829

Moderator: or do you think it's like? Just been a lack on all fronts

295

00:40:38.420 --> 00:40:52.960

A1.1: Honestly, I think, like something like stuff like the study is really good. It's really great like you're asking people honest opinions. I like. I hope that they will be, you know, implemented until making changes. Um,

296

00:40:54.230 --> 00:41:02.499

A1.1: i'm glad that we've started a like a sort of a care team in the past few years. I think that's great. Um, I think more work can be done to like

297

00:41:03.020 --> 00:41:06.920

A1.1: and improve it and make people feel more um

298

00:41:07.520 --> 00:41:09.389

A1.1: sort of uh

299

00:41:09.740 --> 00:41:14.320

A1.1: the less stigma around like availing themselves to those resources.

300

00:41:14.590 --> 00:41:20.469

Moderator: Yeah, Talk more about that, like What do you? What would those recommendations be? What is the Sigma?

301

00:41:20.810 --> 00:41:26.080

A1.1: Yeah. Well, I think there's still a prevailing stigma in academia around

302

00:41:26.360 --> 00:41:31.519

A1.1: um seeking resources for mental health, like

303

00:41:32.100 --> 00:41:49.610

A1.1: especially for like graduate school. I think, like some people per perceive it as weakness like. Oh, you can't cut the you cut it for all the stress and the the hard work, you know, like Phd programs, lots of hard work and stress. And you knew that going into you know.

304

00:41:49.620 --> 00:41:58.020

A1.1: Yeah, just work through it. So I don't think that's a healthy mindset. Um, but I think some of that still there. Um,

305

00:41:59.460 --> 00:42:07.520

A1.1: and you know, like people feeling like they have to work way over time, or they have to work through their vacation.

306

00:42:07.890 --> 00:42:09.049

A1.1: Um!

307

00:42:09.730 --> 00:42:16.970

A1.1: And no one says like you have to do this but that's the it's kind of the unspoken expectation. Um: So

308

00:42:17.870 --> 00:42:18.910

A1.1: yeah,

309

00:42:19.430 --> 00:42:20.459

Moderator: yeah,

310

00:42:20.690 --> 00:42:26.029

Moderator: Do you think it's mostly the stigma that prevents people from using the care, team referrals

311

00:42:26.770 --> 00:42:29.779

Moderator: Or is there anything else. Also that

312

00:42:30.300 --> 00:42:33.679

A1.1: I think that's that's part of it. And

313

00:42:34.100 --> 00:42:53.739

A1.1: also I think we could improve it by like kind of talking in person about it. Um, uh making it feel less like this thing that's out there that we can access through email because we get like an email every month about it. But I don't really hear about it other than that. So um, you know, like me, you

314

00:42:54.650 --> 00:43:01.660

A1.1: I mean like something beyond, just like a monthly email. I think would go a long way

315

00:43:06.170 --> 00:43:07.879

A1.1: that you know

316

00:43:08.720 --> 00:43:17.459

Moderator: I hear you saying that you want something more than just these emails. You want to really feel like it's being addressed in person. And um

317

00:43:18.040 --> 00:43:23.039

Moderator: like more the root of the issue, and not just like, here's an email for a resource. Is that what you're saying?

318

00:43:23.150 --> 00:43:26.169

A1.1: Exactly. Yeah. And like

319

00:43:26.810 --> 00:43:31.880

A1.1: you have to actually care about the well being of the students, and like one of

320

00:43:32.420 --> 00:43:49.519

A1.1: like you have to reach out to them personally, like you can't. An email is not enough, you know. But you know what I mean, like Um, yeah,

Moderator: A place to offer feedback to?

321

00:43:49.560 --> 00:43:50.589

Yeah.

322

00:43:50.850 --> 00:43:54.800

A1.1: Great. But Yeah, I think that's what makes it so hard. It is like

323

00:43:57.970 --> 00:44:00.950

A1.1: you've got to have like people willing to like put themselves out there and be like vulnerable and make people feel like they can talk with you honestly and openly and not be judged for it which is hard to do in a professional setting.

324

00:44:14.770 --> 00:44:15.879

A1.1: Yeah.

325

00:44:15.970 --> 00:44:17.660

A1.1: So yeah,

326

00:44:18.580 --> 00:44:19.580

yeah,

327

00:44:19.790 --> 00:44:20.790

Moderator: agreed

328

00:44:21.530 --> 00:44:23.969

Moderator: Um. Any other thoughts.

329

00:44:31.670 --> 00:44:47.479

Moderator: So I think I've hit the hype once. Um, yeah, Great? Well, thank you for uh participating in this study Uh: your input. Is very valuable to our community. And i'm sure the findings of the study will help to inform and support future. Well-being efforts

330

00:44:47.490 --> 00:45:00.000

Moderator: um If you happen to think of anything else you'd like to share. Please use the survey link. Um. It's included in the recruitment email. You received um, and you'll be able to anonymously provide additional feedback. Um, should you choose to?

331

00:45:00.040 --> 00:45:10.519

A1.1: Great? Well, thank you. Um, Thank you so much. You guys are doing this. So yeah, thanks. Yeah. Have a great rest of your day. Me, too. Thanks, bye,
